# Systematic literature review of burden of illness in chronic inflammatory demyelinating polyneuropathy (CIDP)

**DOI:** 10.1007/s00415-020-09998-8

**Published:** 2020-06-24

**Authors:** Luis Querol, M. Crabtree, M. Herepath, E. Priedane, I. Viejo Viejo, S. Agush, P. Sommerer

**Affiliations:** 1 Institut de Recerca Biomèdica Sant Pau, Barcelona, Spain; 2 Huron Consulting Group, London, UK; 3 Optimal Access Life Science Consulting Limited, Swansea, UK; 4 CSL Behring, Hattersheim, Germany

**Keywords:** CIDP, Burden, QoL, Epidemiology, Treatment, Cost

## Abstract

**Background:**

Chronic inflammatory demyelinating polyneuropathy (CIDP) is a rare neurological disorder characterised by muscle weakness and impaired sensory function. The present study provides a comprehensive literature review of the burden of illness of CIDP.

**Methods:**

Systematic literature search of PubMed, Embase, and key conferences in May 2019. Search terms identified studies on the epidemiology, humanistic burden, current treatment, and economic burden of CIDP published since 2009 in English.

**Results:**

Forty-five full texts and nineteen conference proceedings were identified on the epidemiology (*n* = 9), humanistic burden (*n* = 7), current treatment (*n* = 40), and economic burden (*n* = 8) of CIDP. Epidemiological studies showed incidence and prevalence of 0.2–1.6 and 0.8–8.9 per 100,000, respectively, depending on geography and diagnostic criteria. Humanistic burden studies revealed that patients experienced physical and psychosocial burden, including impaired physical function, pain and depression. Publications on current treatments reported on six main types of therapy: intravenous immunoglobulins, subcutaneous immunoglobulins, corticosteroids, plasma exchange, immunosuppressants, and immunomodulators. Treatments may be burdensome, due to adverse events and reduced independence caused by treatment administration setting. In Germany, UK, France, and the US, CIDP economic burden was driven by direct costs of treatment and hospitalisation. CIDP was associated with indirect costs driven by impaired productivity.

**Conclusions:**

This first systematic review of CIDP burden of illness demonstrates the high physical and psychosocial burden of this rare disease. Future research is required to fully characterise the burden of CIDP, and to understand how appropriate treatment can mitigate burden for patients and healthcare systems.

**Electronic supplementary material:**

The online version of this article (10.1007/s00415-020-09998-8) contains supplementary material, which is available to authorized users.

## Introduction

Chronic inflammatory demyelinating polyneuropathy (CIDP) is a rare, immune-mediated disorder in which an aberrant immune response causes demyelination and axonal damage of the peripheral nerves [1, 2]. The exact aetiology of CIDP remains unknown [2, 3]. Patients experience progressive weakness, impaired sensory function in the legs and arms, loss of deep tendon reflexes (areflexia), and fatigue [1, 4, 5].

CIDP is a long-term condition with a variable course that can be relapsing–remitting, stepwise progressive, or gradually progressive [2, 3]. Axonal damage occurs with further disease progression, resulting in worsening symptoms [6]. Patient symptom burden can be assessed using a range of functional outcomes that predominantly focus on physical burden, functional impairment, disability, and an impaired ability to perform activities of daily living [7–9]. Impairment assessment tools include the Inflammatory Neuropathy Cause and Treatment (INCAT) scale and the inflammatory Rasch-Built Overall Disability Scale (I-RODS). INCAT assesses physical function from 0 (no functional impairment) to 10 (unable to purposefully move the limbs) [8–10]. I-RODS is a 24-item scale; each item represents a daily activity (e.g., reading a newspaper and running), scored from 0 (impossible to perform) to 2 (easy to perform) [10]. Despite the existence of such tools, to date, the disease burden and patient impact of CIDP has not been well defined.

European Federation of Neurological Societies/Peripheral Nerve Society (EFNS/PNS) guidelines provide recommendations on CIDP treatments, with the goal of reducing symptoms and, if possible, maintaining long-term remission [11, 12]. Treatment with intravenous immunoglobulin (IVIG) or corticosteroids is recommended in patients with moderate to severe disability. If IVIG and corticosteroids are ineffective, plasma exchange should be considered. If the response is inadequate or the required drug maintenance dose is high, combination treatments of either immunosuppressants or immunomodulators should be considered. No recommendations are provided on long-term management, due to lack of evidence [11]. Moreover, treatments may be associated with adverse events (AEs) or reduced patient independence [3, 13]. For example, corticosteroids are associated with long-term tolerability challenges, while IVIG needs to be regularly administered in a clinical setting or at home under nurse supervision [13–16].

Treatment of CIDP is complicated by challenges in diagnosis [17]. The disease can present with differing symptoms, and there are at least 15 sets of diagnostic criteria that describe CIDP and its variant forms [1, 6]. Varying presentation of CIDP and misinterpretation of nerve conduction studies result in a high rate of misdiagnosis [6, 18]. This can result in inappropriate treatment, as patients with CIDP may be misdiagnosed with other polyneuropathies, such as anti-myelin associated glycoprotein (MAG) neuropathy or polyneuropathy of POEMS syndrome (polyneuropathy, organomegaly, endocrinopathy, M protein, and skin changes), which require different treatments than CIDP [17].

There are significant challenges in characterising the burden of illness for rare diseases such as CIDP, and evidence can be limited [19]. Identifying and subsequently recruiting patients to studies are challenging; low sample sizes may impair generalisability and statistical powering [19]. To date, no publication has comprehensively reviewed the burden of illness of CIDP with respect to epidemiology, humanistic burden, current treatments, and economic burden. The current study provides a systematic review of the literature to characterise the burden of the disease and identify key areas for future research.

## Methods

### Search strategy

Keywords related to the study topics were used to search relevant research articles in the MEDLINE (including In-process) and Embase databases. Only articles including human data and published in English from May, 2009 to May, 2019 were included. Separate search terms were used for each review category. The search algorithms, limits, and number of hits obtained are summarised in Online Resource 1.

To ensure inclusion of more recent studies that had not yet been published in peer-reviewed journals, we reviewed non-peer-reviewed articles from the following neuromuscular conferences and conferences relevant to health economic and outcomes research, published between 2017 and 2019: International Society for Pharmacoeconomics and Outcomes Research (ISPOR) conference, International Congress on Neuromuscular Diseases (ICNMD), and Annual Meetings of the Peripheral Nerve Society (PNS). It was assumed that any conference outputs prior to these dates would have been published in a peer-reviewed journal at the time that the searches were conducted.

### Study selection

Study selection was completed through two levels of study screening: abstract screening and full-text screening. Abstract screening was performed in the web-based software platform, abstrackr^Beta^. Eligible studies were identified by one reviewer for inclusion in the study according to the predefined inclusion and exclusion criteria (Table 1). Studies had to fulfil all inclusion criteria and none of the exclusion criteria at each level of screening to be included in the data extraction stage. Full articles were retrieved for abstracts deemed relevant, and a full article review determined the final inclusion or exclusion of a study, based on the predetermined criteria. Rejected studies at the abstract and full-text levels were reviewed by a second reviewer and any discrepancies, in terms of rejection decision or reason, were resolved by consensus between the two reviewers. Information from the included full texts was extracted into a data extraction MS Excel table. Data relevant to the inclusion criteria were extracted by one reviewer and quality checked by a second reviewer. All included publications were assessed to ensure that there was no duplicate reporting of data.

**Table 1:**
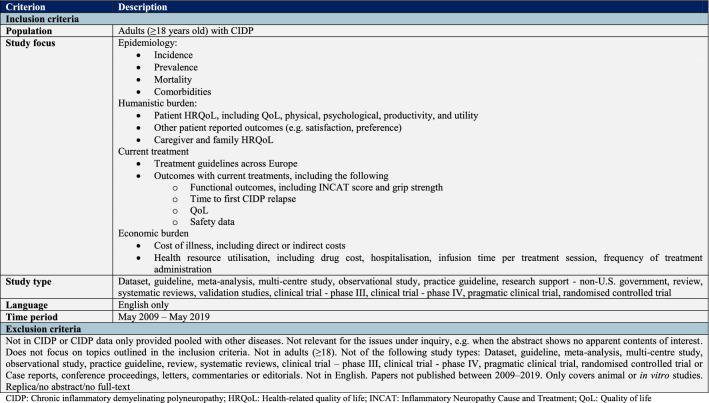
Study inclusion and exclusion criteria

*CIDP* chronic inflammatory demyelinating polyneuropathy, *HRQoL* health-related quality of life, *INCAT* inflammatory neuropathy cause and treatment, *QoL* quality of life

## Results

**Fig. 1:**
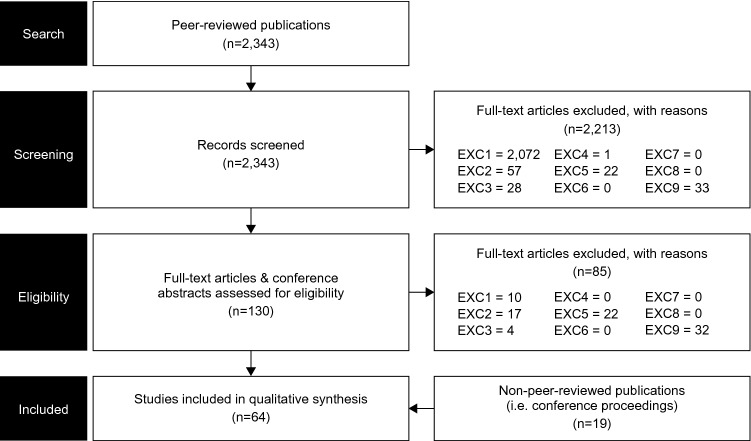
PRISMA diagram. EXC1: not in CIDP OR: CIDP data only provided pooled with other diseases; EXC2: not relevant for the issues under inquiry, e.g., when the abstract shows no apparent contents of interest; EXC3: does not focus on topics outlined in the inclusion criteria (see Table 1.) EXC4: not in adults (≥ 18); EXC5: not of the following study types: dataset, guideline, meta-analysis, multi-centre study, observational study, practice guideline, review, systematic reviews, clinical trial—phase III, clinical trial—phase IV, pragmatic clinical trial, randomised-controlled trial OR: case reports, conference proceedings, letters, commentaries or editorials; EXC6: not in English; EXC7: papers not published between 2009 and 2019; EXC8: only covers animal or in vitro studies; EXC9: replica/no abstract/no full text

A total of 2343 studies were identified from the MEDLINE and Embase databases. After abstract screening, 130 full texts were assessed, of which 45 articles met the inclusion criteria and were taken forward to data extraction. After inclusion of 21 abstracts identified from the ICNMD, PNS, and ISPOR conferences, 66 articles were deemed to have met the inclusion criteria and were included in the analysis. Included articles were categorised by topic: epidemiology (*n* = 9), humanistic burden (*n* = 7), current treatments (*n* = 42), and economic burden (*n* = 8). Figure 1 illustrates the study selection process.

### Epidemiology

Epidemiology publications were either based on real-world studies (*n* = 6, 67%)—typically derived from patient clinical databases—or based on literature reviews (*n* = 3, 33%) [1, 4–7, 20–23]. Real-world studies reported epidemiology from Italy (*n* = 2), England (*n* = 1), Iceland (*n* = 1), and The Netherlands (*n* = 1) [7, 20–23]; the geography of one study was not reported [5]. Literature reviews reported on multinational epidemiology data, including countries within the Middle East, Western and Eastern Africa, Europe, North and South America, and East Asia [1, 4, 6].

Incidence and prevalence data identified in the systematic review are presented in a graphical summary in the Online Resource 2. CIDP incidence ranged from 0.2 to 1.6 per 100,000 persons per year [1, 4–6], while prevalence was in the range of 0.8–10.3 per 100,000 persons [1, 4, 6, 20]. Incidence and prevalence rates varied with age, gender, and diagnostic criteria [1, 4–7, 20–23]. CIDP prevalence was higher in males than females and increased with age [1, 4, 5], with one study reporting a mean patient age of 57 years [5]. Mahdi-Rogers et al. 2014 reported that the mean age of onset of CIDP ranged from 48 to 59.6 years [20]. Pooled crude prevalence data from studies using the American Academy of Neurology (AAN) diagnostic criteria were lower than those studies using the EFNS 2006 criteria (1.59 and 3.67 per 100,000, respectively) [1].

Both studies investigating patient survival reported similar mortality to the general population [5, 6]. Ryan et al. 2018 reported on one of the first cohorts of patient with CIDP from the USA (study conducted in 1975, *n* = 53), with a mortality of 10% over 7.5 years [6]. A more recent study by Hafsteindottir et al. 2016 reported a standardised mortality ratio of 0.9 for Iceland (determined using life tables), which does not vary from mortality rate of the general population [5].

No clear risk factors for a diagnosis of CIDP were reported, although data highlighted potential associations with autoimmune disease, diabetes, hypertension, and antecedent infection [6, 7, 20, 21]. Kuitwaard et al. 2009 showed that 5% of the study CIDP cohort (*n* = 76) were diagnosed with a common autoimmune disease (e.g., thyroid disorder and rheumatic disorders), which was higher than the frequency in the general population [7]. Between 9.9 and 12.6% of patients with CIDP had diabetes and there was a ninefold higher prevalence of CIDP in patients with diabetes, compared with the general population [6, 20]. Despite this, studies could not determine whether diabetes was a risk factor for CIDP or if diabetes was more common in patients with CIDP [6, 20]. Hypertension was reported in 17% of Italian patients in a study by Doneddu et al. 2017 [21]. An antecedent infection, or vaccination, preceded a diagnosis of CIDP in 27% of patients [21].

### Humanistic burden

All humanistic burden studies were real-world studies, and included data derived from analyses of trial patient cohorts, clinical patient examinations, or surveys of patient member organisations [7–9, 24–27]. The studies included populations from the UK (*n* = 2), Netherlands (*n* = 1), Germany (*n* = 1), and multinational (*n* = 1) studies, while the geographies of two studies were not reported [7–9, 24–27]. Humanistic burden studies reported on the patient impact of CIDP on health-related quality of life (HRQoL), functioning, psychosocial welfare, productivity, pain, and fatigue [7–9, 24, 25].

CIDP has a substantial physical impact, with patients reporting pain, fatigue, and impaired physical functioning [7–9, 24–27]. Kuitwaard et al. 2009 assessed pain and fatigue, using the Numeric Pain Rating Scale (NPRS) and Fatigue Severity Scale (FSS) scores (*n* = 76), reporting that 17 and 74% of patients with CIDP reported severe pain and severe fatigue, respectively [7]. A German study (*n* = 108) reported a mean INCAT total score of 3.3 [9]; patients with a score of 3.3 may experience impairments in completing daily activities (e.g., unzipping, using a knife and fork) or require some support to walk outdoors [10].

The impact of CIDP may also extend beyond the physical burden of the disease to depression [7, 9, 28]. Two publications reported that 9 and 12.1% of patients had depression, based on hospital anxiety and depression scale (HADS) and the Beck Depression Inventory version II (BDI-II) scores in The Netherlands and Germany, respectively [7, 9]. Cognitive function is, however, unaffected; a German study (*n* = 107) reported Mini-Mental State Examination (MMSE) scores of 28.8 for patients with CIDP [9], where MMSE scores 0–17 and 24–30 reflect severe and no cognitive impairment, respectively [28].

**Fig. 2:**
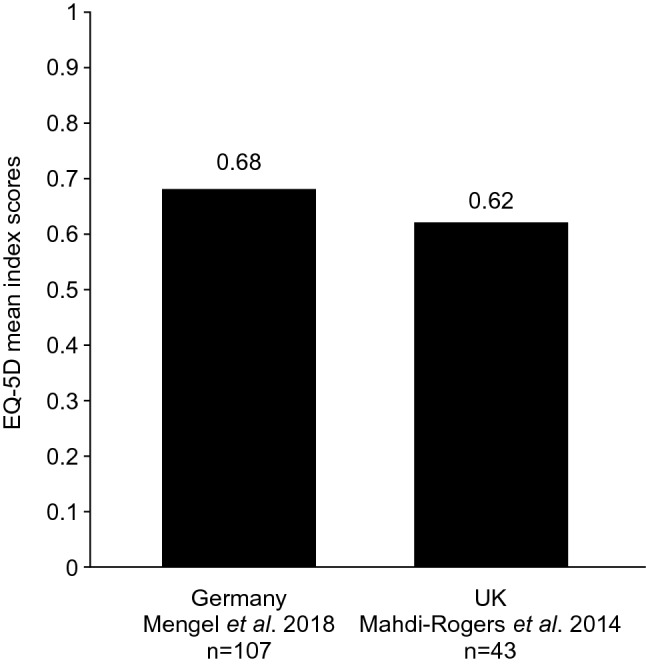
EQ-5D mean index scores of patients with CIDP [9, 25]. *CIDP* chronic inflammatory demyelinating polyneuropathy, *EQ*-*5D* EuroQoL-5D

**Fig. 3:**
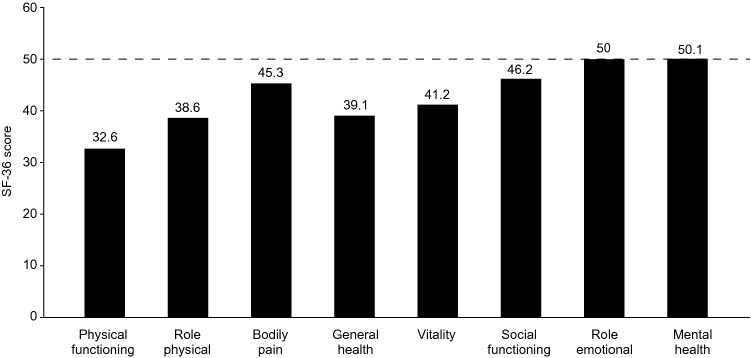
SF-36 scores in a Dutch cohort of patients with CIDP Adapted from Kuitwaard et al. 2009 [7]. The dashed line reflects the normative value of SF-36 by healthy patients: 50 points. Sample size; *n* = 76. *CIDP* chronic inflammatory demyelinating polyneuropathy, *SF*-*36* Short Form-36

The physical and mental manifestations of CIDP impair patient well-being and quality of life [7–9, 25]. EuroQol-5D (EQ-5D) scores among patients with CIDP were 0.62 and 0.68 for the UK and Germany, respectively (Fig. 2) [9, 25]. Draak et al. 2014 demonstrated that lower EQ-5D for patients with CIDP was significantly associated with impaired patient physical function [8]. The *R*^2^, or fraction of variance explained by independent variables from a regression model, for the relationship between the EQ-5D and the INCAT Overall Neuropathy Limitations Scale (INCAT-ONLS; *R*^2^ = 0.30–0.32) and I-RODS (*R*^2^: 0.42) were significant (*p* < 0.0001) [8]. A study of Dutch patients with CIDP (*n* = 76) also identified that patients scored below normative 36-Item Short Form Survey Instrument (SF-36) scores (50) for all the domains of the physical component and two of the domains of the mental component (vitality and social functioning) (Fig. 3) [7]. The role emotional and mental health domains were, however, similar to normative scores [7]. The following World Health Organization Quality of Life Brief Version (WHO-QoL BREF) questionnaire data were also reported on a scale from 0 to 100 in a German population: global (47.2), physical health (55.4), psychological health (56.1), social relationships (66.2), and environmental (73.2); no comparison to normal population scores was provided [9].

Two studies reported that premature retirement due to CIDP occurred among 14–28% of patients [9, 26]. Bjelica et al. 2018 reported that the odds of depression, based on BDI-II, and fatigue, based on FSS, were, respectively, 12.2 and 8.2 times greater in retired patients with CIDP, compared with non-retired CIDP patients [26].

### Guidelines and current treatment

Publications on current treatments included clinical trials (*n* = 23, 60%), real-world studies (*n* = 13, 31%), literature reviews (*n* = 3, 7%) and a combined real-world and clinical trial (*n* = 1, 2%). The majority of clinical trials (including the combined real-world and clinical trial) were comparative studies, with either a placebo arm (*n* = 10, 38%) or an active comparator (*n* = 9, 35%). A minority of real-world studies focused on a single treatment (*n* = 4, 31%); the remaining reported on multiple treatments (*n* = 9, 69%). Sample sizes for the real-world studies, which included analyses of databases and patient examinations, ranged from 13 to 432, while the clinical trial sample sizes ranged from 27 to 265. No treatment guidelines were identified.

Studies were highly heterogeneous regarding intervention, comparator, time point, and methodology. Definition of “treatment response” varied across studies, and included predefined score increases in functional outcome scales (e.g., Rankin scale), treating physician assessment, and measurement of strength (e.g. grip strength) [15, 29, 30]. Review findings are summarised below for treatment response rate, patient preference, and treatment tolerability. Further data are provided in Online Resource 3.

Although many patients responded to corticosteroid therapy (48–83% response rate, across nine studies), safety concerns were associated with their long-term use [12, 15, 16, 31–35]. Common AEs included hypertension, diabetes mellitus de novo, glaucoma, depression, cushingoid appearance, and gastrointestinal complaints [15, 16]. Consequently, many patients in high-income countries receive IVIG, which has similar efficacy and is associated with fewer AEs [16].

IVIG generally achieved high rates of response to treatment (44–91% response rate, across 11 studies), relative to placebo [12, 30–32, 35–43]. Where comparative data were reported, IVIG increased the proportion of patients with CIDP who responded to treatment, relative to comparators, including corticosteroids and plasma exchange [12, 29, 31]. Markvardsen et al. 2013 reported that during long-term IVIG therapy, intravenous access can become difficult due to obliteration of the veins and may necessitate catheterisation of the external jugular vein in some patients [14].

Subcutaneous immunoglobulins (SCIG) provide an alternative route of administration that may ameliorate some of the challenges associated with intravenous administration of immunoglobulins [14, 44–48]. Three studies reported on the clinical stability of patients with CIDP after commencing SCIG, with all studies finding that the majority of patients were clinically stable or improved (83–100%; *n* = 13–245) [39, 47, 48]. The largest trial on SCIG treatment (PATH, *n* = 172, placebo-controlled) found the proportion of patients with a CIDP relapse or who were withdrawn from the trial for any other reason which was 63% for patients in the placebo group, 39% for patients in the low-dose SCIG group, and 33% for patients receiving high-dose SCIG [46].

Studies on SCIG patient-reported outcomes (PRO) data predominantly reported on ease of use or patient preference [14, 44, 46, 49]. In Van Schaik et al. 2017, 88% (*n* = 172) of patients reported that learning the SCIG administration technique was easy [46]. Three studies reported on patient preference, demonstrating that 53–88% of patients preferred SCIG to IVIG [14, 44, 46]. Reasons for preferring SCIG included gaining independence, ease of use, increased flexibility during daily life, more stable muscle performance, and milder side effects, and that the treatment was time saving compared with IVIG [44, 46]. Van Schaik et al. 2017 reported that an open-label prospective study showed a significant reduction in the severity and frequency of headaches and nausea after SCIG infusions compared with IVIG infusions [46]. Cocito et al. 2018 reported that SCIG use was associated with improved QoL and fewer systemic AEs compared to IVIG in observational studies [44].

Supplementing IVIG with a concomitant immunosuppressant may be an effective strategy for improving response rates or reducing IVIG dosages [50, 51]. Querol et al. 2013 reported that the percentage of therapy responders was higher among patients who received concomitant immunosuppressant treatment than those who received IVIG only (71.4 versus 44.0%; OR 3.18) [42]. Mahdi-Rogers et al. 2009 showed that methotrexate reduced mean weekly dose of IVIG or SCIG in 52% (*n* = 27) of patients compared with a 44% (*n* = 32) reduction in the placebo group [50].

Plasma exchange may provide an alternative therapy for patients who do not respond to IVIG and corticosteroids [12, 31–34]. Although 48–81% of patients across studies responded to plasma exchange [12, 31–34], comparative data suggest that fewer patients achieve a response for plasma exchange compared with IVIG [12, 31–34]. In a study of 16 patients with CIDP, plasma exchange was associated with difficulty in accessing veins and deficit of blood coagulation factors [31]. In the same study, a higher proportion of patients experienced AEs with plasma exchange (25%) than with corticosteroids (13%) or IVIG (4%) [31].

Publications on the use of immunomodulators (oral fingolimod and intramuscular interferon ß-1a) for CIDP indicated no improvement in efficacy compared with placebo [52–54].

Limited data were available on ability to predict treatment response; only one study explored the ability to predict treatment response between diagnostic criteria (EFNS, AAN, INCAT, and Saperstein criteria) [35]. Patients responded to IVIG treatment to a similar extent (66–91%, *n* = 9–22) irrespective of the diagnostic criteria used, although the EFNS 2010 criteria were the most diagnostically sensitive [35].

### Economic burden

Eight publications reported on the economic burden of CIDP [9, 24, 25, 55–59]. The majority of studies reported data from European countries (*n* = 5, 62.5%), including the UK (*n* = 2), France (*n* = 2), and Germany (*n* = 1) [9, 24, 25, 55, 56, 58]. Three studies reported data from the USA [56, 57, 59]. Most studies had a sample size of less than 150 patients (range 15–1580) [9, 24, 25, 55–59]. A full summary of cost data identified in the review is provided in Online Resource 4.

CIDP cost of illness varied across geographies, from £22,086 (2007 costs) annually per patient in the UK, through €47,823 (2017 costs) and €45,000 (2013 costs) annually per patient in France and Germany, respectively, to $116,330 (costs converted to USD 2016 values) over 2 years in the USA (Online Resource 4) [9, 25, 55, 56].

**Fig. 4:**
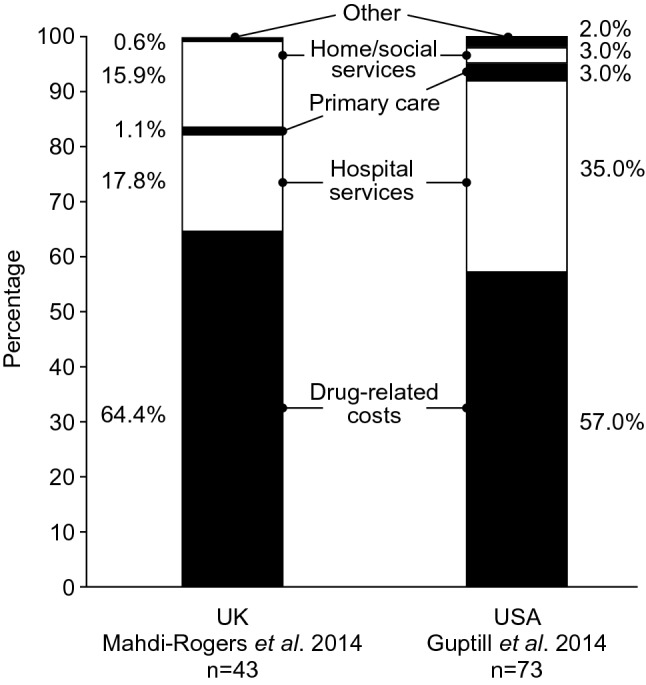
Distribution of CIDP direct costs by cost components in the UK and USA [25, 57]. USA costs were calculated on a 2-year basis [57], whereas UK costs are based on an annual estimate [25]

Drug-related costs were the main drivers of direct CIDP expenditure, followed by costs associated with hospital services (Fig. 4) [25, 57]. In the UK, IVIG and other treatments represented the largest proportion of total costs (64.4%), followed by hospital services (17.8%) [25]. Similarly, in the US, drug-related costs represented 57% of total costs, followed by inpatient and outpatient hospital services (35%) [57].

CIDP was also associated with indirect costs, driven predominantly by impaired productivity [9, 25]. In Germany, mean indirect 3-month costs were €1910 per patient, comprising 17% of total CIDP costs [9]. Indirect costs included premature retirement (€945), disability (€276), unemployment (€189), sick leave (€283), and reduction of labour time (€217) [9]. In the UK, loss of productivity was estimated to cost £5815 per patient per year, and projected to cost the UK economy a total of £9.7 M per year, accounting for 26% of the total cost of illness (Online Resource 4) [25].

Total costs and resource use varied depending on the setting of IVIG administration and other factors, such as patient well-being and physical functioning [9, 55]. Le Masson et al. 2018 demonstrated a difference between home and in-hospital therapy (€62,592 versus €106,867 total year cost, respectively, *p* < 0.0001) associated with hospital admission and patient care costs [55]. In the US, the majority of IVIG infusions occurred at home (72%), followed by physician offices (16%) and outpatient hospitals (11%) [57]. In France, however, 43%, 49%, and 8% of IVIG infusions require full hospitalisation, day care, and home care, respectively [58].

In Germany, increased direct and medication costs were significantly associated with functional disability (measured by INCAT) and clinically relevant depressive symptoms (measured by BDI-II), *p* < 0.005 for both [9]. The presence of the depressive symptoms was also associated with increases in medication costs [9]. Improved WHO-QoL BREF and functional disability scores were associated with reduced indirect (*p* < 0.011) but not direct costs [9].

## Discussion

The current review provides the first systematic assessment of the burden of illness of CIDP. The findings establish that CIDP is a rare disease, associated with impaired HRQoL, particularly relating to physical well-being, and an economic burden. Patients with CIDP may also experience burden due to treatment. Long-term steroid use is associated with a high risk of AEs, and parenteral therapies, such as IVIG, SGIG and plasma exchange, are associated with burden of administration and potential impact on patient independence [12, 16].

Epidemiology studies confirmed that CIDP is a rare disease (incidence: 0.2–1.6/100,000 persons per year; prevalence: 0.8–10.3 per 100,000 persons) [1, 4, 6, 20]. The incidence and prevalence data varied, likely arising from variations in study sample size (*n* = 19–360) and diagnostic criteria. Low sample sizes are common when identifying and recruiting patients with rare diseases [19]. Through combining datasets, meta-analyses can mitigate some of the challenges posed by low sample size and diagnostic variation [60]. The study by Broers et al. 2019 used this approach, reporting incidence and prevalence for CIDP of 0.33 per 100,000 person-years and 2.81 per 100,000 persons, respectively, which places CIDP among the rarest neuropathies [1, 4]. In contrast, one of the most common chronic polyneuropathies, diabetic polyneuropathy, is estimated to have a prevalence rate of 200–600/100,000 [4]. Moreover, diagnostic errors, such as over-reliance on subjective patient-reported outcomes and diverse electrophysiologic interpretations of demyelination, contribute to diagnostic uncertainty and may impact epidemiology findings [18]. Misdiagnosis can occur and patients may be treated inappropriately as a result [17, 18, 37]. Clinicians may use supportive measures, such as patient treatment response to confirm a CIDP diagnosis, to improve diagnostic accuracy [3, 11, 12, 37]. This may, however, contribute to misdiagnosis when treatment response is not evaluated appropriately [18]. Further research is needed to fully characterise the impact of false-positive and false-negative diagnoses.

CIDP was associated with impaired patient well-being, due to the physical impact of CIDP and, in some cases, the presence of mental health conditions. Patients with CIDP had EQ-5D index scores of 0.68 in Germany and 0.62 in the UK, substantially below normative values for these countries (Germany: 0.90; UK: 0.86) [9, 25, 61]. For comparison, EQ-5D mean scores of 0.77 and 0.57 have been reported for patients with multiple sclerosis in Germany and the UK, respectively [62, 63]. EQ-5D scores were also significantly associated with physical function scores (INCAT-ONLS and I-RODS), demonstrating the importance of physical function in the well-being of patients with CIDP [8]. The physical impact of CIDP can prevent patients from completing daily activities and walking outdoors without support [9]. However, physical functioning was implicated in 30–42% of the observed changes in HRQoL in patients with CIDP [8], showing that, while physical disability is linked to impaired QoL, there are additional contributing factors. Patients with CIDP also reported higher rates of depression in The Netherlands and Germany, compared with the general population averages reported by the European Brain Council and the European College of Neuropsychopharmacology (9–12.1 versus 5.9%) [7, 9, 64]. This may reflect the impact that chronic disabling conditions have on mental health [65].

Treatment setting, mental health, and physical functioning were associated with an increased economic burden in CIDP; however, only eight studies on economic burden were identified and results need to be interpreted with caution. While treatment costs were the primary cost drivers, research in USA and France indicates that the cost of treatment of CIDP reduces if patients can receive IVIG treatment at home rather than in a hospital setting [55]. Depression and functional disability were also identified as important predictors of direct costs [9]. Improving depression and functional disability may reduce costs in CIDP, although further research is required to establish the nature of this relationship. These findings cannot be generalised beyond the individual country settings, however, as healthcare systems vary extensively across geographies. Publications were limited to English language studies, impairing identification of multinational studies, and economic data from only four countries were represented in the review.

The physical and psychological impact of CIDP is implicated in productivity losses and indirect costs. Early retirement was reported among patients with CIDP [9, 26]. The odds of fatigue and depression were higher in retired patients with CIDP, versus non-retired CIDP patients (8.2 and 12.2, respectively) [26], and the majority of patients with CIDP experience severe fatigue and depression rates that are greater than for the general population [7, 9, 64]. Functional disability (measured by INCAT) and impaired HRQoL were also predictors of higher indirect costs in Germany, due to premature retirement, disability, unemployment, sick leave, and reduced labour time [9]. This impact on productivity can result in substantial economic costs; for example, productivity losses due to CIDP may result in 9.7 M annual economic cost for the UK (estimated through value of lost wages) [25]. Similarly, in Germany premature retirement was the second highest cost element of all cost categories when assessing the costs associated with CIDP, following cost of medication [9].

Current therapies can improve patient well-being, but are also associated with tolerability issues and challenges around route of administration [13]. IVIG and corticosteroids are both effective in achieving a treatment response, reflecting EFNS/PNS recommendations to use as the first-line therapies [11]. However, long-term use of corticosteroids has been associated with serious side effects in patients with CIDP, such as hypertension, Cushingoid appearance, and gastrointestinal complaints [3, 15, 16]. Long-term use of corticosteroids is typically avoided where possible in other diseases; for example, corticosteroid-sparing approaches (prescribing alternative treatment strategies to reduce corticosteroid dose) are considered important in other autoimmune diseases, such as Crohn’s disease and uveitis [66, 67]. Although the majority of studies reporting comparative data for IVIG reported a greater response rate relative to other therapies [12, 15, 16, 31–35], IVIG may be burdensome for patients [13, 14]. IVIG requires venous access, patient monitoring, and administration in a clinical setting or through home nursing services, which may contribute to increases in healthcare costs and reductions in patient independence [13, 14].

SCIG has been developed as an alternative to IVIG, providing flexible self-administration at home [13]. While IVIG administration requires a nurse to supervise infusions, SCIG can be administered by the patient or a caregiver without the need for other medical support staff, which may decrease healthcare resource use [13]. Patients managed with SCIG are not dependent on home nursing visits or maintaining proximity to an infusion centre so can travel more freely [13]. Studies suggest that SCIG has similar efficacy to IVIG, and confirm that patients prefer SCIG due to reductions in AEs and improved independence relative to IVIG [14, 44, 46, 49]. A previous meta-analysis of efficacy and safety of SCIG versus IVIG consolidates these findings, revealing that the relative risk of systemic AEs (e.g., headache and fever) was reduced by 28% with SCIG versus IVIG (95% CI 0.11–0.76), while effectiveness was similar between the two groups [6]. As CIDP is a chronic disease that is not associated with increased mortality [5, 6], patients require treatments that are appropriate for long-term use. SCIG may benefit patients’ long-term disease management, through decreasing systemic AEs and improving patient independence compared with alterative treatment options.

EFNS/PNS guidelines (2010) do not provide guidance on long-term management of CIDP, nor on SCIG, as evidence on these topics was insufficient at the time of guideline development [11]. Treatment selection needs to consider a range of variables, such as severity, health status, tolerability, and contraindications, which may present challenges to healthcare professionals [68]. Treatments further need to be reviewed on an ongoing basis to ensure that treatments are appropriate and avoid unnecessary burden. For example, it is recommended that IVIG is periodically reduced or withdrawn to avoid excessive costs, while corticosteroids should be reviewed or withdrawn to avoid AEs [15, 39]. CIDP subtype and varied diagnostic criteria may also be important in treatment decisions; however, the evidence is unclear. Patient populations varied extensively between the publications captured in the current review and the heterogeneity presents challenges to drawing robust conclusions [35]. The limited evidence available suggests that treatment outcomes do not vary based on diagnostic criteria used to determine the presence of CIDP; however, further research is required to establish this and the role of subpopulations in determining response to treatment. Further research incorporating SCIG as a long-term treatment option for CIDP may also inform future disease management strategies.

### Limitations

There may be additional publications not identified in this review that are important for characterising the burden of disease in CIDP. For example, relevant data may have been published that were excluded as a result of the limits to the study type and publication dates. Searches were restricted to English language publications, which may have excluded relevant studies published in other languages.

No studies were identified related to treatment guidelines in the current review; however, the EFNS/PNS guidelines on management of chronic inflammatory demyelinating polyradiculoneuropathy” were published within the dates in scope [11]. This publication was not captured in this review, as the search algorithm was limited to “adults” and these guidelines included paediatric considerations and so were not indexed to “adults” within the searched databases.

Limitations characteristic of CIDP were identified, such as low number of relevant publications and low sample sizes in studies. The limited number of publications available on epidemiology, humanistic burden, and economic burden impairs the current assessment of the burden of disease. The low number of publications identified reflects the rarity of the disease, as research into rare disease may face greater challenges in recruiting patients and obtaining funding, because more common diseases have a greater economic impact [19]. The majority of studies across categories included fewer than 150 patients, reflecting the challenges in patient identification and recruitment in rare diseases [19]. Low sample sizes introduce challenges for deriving insights from the data, such as poor generalisability and lack of statistical powering [19].

## Conclusion

This review provides the first systematic assessment of the burden of illness across all aspects of CIDP. The findings establish that CIDP is among the rarest neuropathies and is associated with substantial patient burden. Further research is required to fully characterise the burden of CIDP, particularly the physical impact on patients, and to understand how appropriate treatment can support mitigating disease burden for patients and healthcare systems.

## Electronic supplementary material

Below is the link to the electronic supplementary material.

Supplementary material 1 (PDF 111 kb)

Supplementary material 2 (PDF 150 kb)

Supplementary material 3 (PDF 471 kb)

Supplementary material 4 (PDF 226 kb)
