# Supplementary material for: Systematic literature review of burden of illness in chronic inflammatory demyelinating polyneuropathy (CIDP)
Source: J Neurol. 2020 Jun 24;268(10):3706–16. doi: 10.1007/s00415-020-09998-8 (PMC8463372; doi:10.1007/s00415-020-09998-8)
Supplement: Supplementary file 1 — Supplementary material 1 (PDF 111 kb) [file 415_2020_9998_MOESM1_ESM.pdf]

Electronic Supplementary Material

Online Resource 1

Table 1. MEDLINE & EMBASE Search Terms

Search	Query	Items found
**Epidemiology**		
#1	(((((("chronic inflammatory demyelinating polyneuropathy"[Title/Abstract]) OR CIDP[Title/Abstract]) OR “chronic relapsing polyneuropathy”[Title/Abstract]) OR CRP[Title/Abstract]) OR “chronic inflammatory demyelinating polyradiculoneuropathy”[Title/Abstract]) NOT "CODP"[Title/Abstract]) NOT "Hs-CRP"[Title/Abstract]) NOT "C-reactive protein"[Title/Abstract] AND (epidemiology[MeSH Terms] OR epidemiolog*[Title/Abstract] OR incidence[Title/Abstract] OR prevalence[Title/Abstract] OR survival[Title/Abstract] OR mortality OR "risk factor" OR comorbi* OR “natural history ”)<br><br>**Limits:** English, humans, adults, 10 years, dataset, guideline, meta-analysis, multi-center study, observational study, practice guideline, research support - Non-U.S. Gov't, review, systematic reviews, validation studies	994
**Humanistic burden**		
#2	((((((("chronic inflammatory demyelinating polyneuropathy"[Title/Abstract]) OR CIDP[Title/Abstract]) OR “chronic relapsing polyneuropathy”[Title/Abstract]) OR CRP[Title/Abstract]) OR “chronic inflammatory demyelinating polyradiculoneuropathy”[Title/Abstract]) NOT "CODP"[Title/Abstract]) NOT "Hs-CRP"[Title/Abstract]) NOT "C-reactive protein"[Title/Abstract] AND ("quality of life"[MeSH Terms] OR Outcomes[Title/Abstract] OR “quality of life"[Title/Abstract] OR QoL[Title/Abstract] OR HRQoL[Title/Abstract] OR patient-reported[Title/Abstract] OR productiv*[Title/Abstract] OR (humanistic[Title/Abstract] OR disease[Title/Abstract] OR patient[Title/Abstract] OR caregiver[Title/Abstract] OR psychological[Title/Abstract] OR psychosocial[Title/Abstract] OR physical[Title/Abstract] AND burden[Title/Abstract]))	556

	**Limits:** English, humans, adults, 10 years, dataset, guideline, meta-analysis, multi-center study, observational study, practice guideline, research support - Non-U.S. Gov't, review, systematic reviews, validation studies	
**Guidelines & current treatment**		
#3	((((((("chronic inflammatory demyelinating polyneuropathy"[Title/Abstract]) OR CIDP[Title/Abstract]) OR “chronic relapsing polyneuropathy”[Title/Abstract]) OR CRP[Title/Abstract]) OR “chronic inflammatory demyelinating polyradiculoneuropathy”[Title/Abstract]) NOT "CODP"[Title/Abstract]) NOT "Hs-CRP"[Title/Abstract]) NOT "C-reactive protein"[Title/Abstract] AND ((practice guidelines as topic[MeSH Terms] OR guideline*[Title/Abstract] OR guidance[Title/Abstract] OR “European Federation of Neurological Societies/Peripheral Nerve Society”[Title/Abstract] OR "task force"[Title/Abstract] OR consensus[Title/Abstract] OR corticosteroid[Title/Abstract]) OR (dexamethasone[Title/Abstract] OR methylprednisolone[Title/Abstract] OR prednisone[Title/Abstract] OR prednisolone[Title/Abstract] OR immunoglobulin[Title/Abstract]))<br><br>**Limits:** English, humans, adults, 10 years, dataset, guideline, meta-analysis, multi-center study, observational study, practice guideline, research support - Non-U.S. Gov't, review, systematic reviews, validation studies, clinical trial - phase III, clinical trial - phase IV, pragmatic clinical trial, randomized controlled trial	566
**Economic burden**		
#4	((((((("chronic inflammatory demyelinating polyneuropathy"[Title/Abstract]) OR CIDP[Title/Abstract]) OR “chronic relapsing polyneuropathy”[Title/Abstract]) OR CRP[Title/Abstract]) OR “chronic inflammatory demyelinating polyradiculoneuropathy”[Title/Abstract]) NOT "CODP"[Title/Abstract]) NOT "Hs-CRP"[Title/Abstract]) NOT "C-reactive protein"[Title/Abstract] AND ("Economic burden"[Title/Abstract] OR costs[Title/Abstract] OR "healthcare utili*"[Title/Abstract] OR "resource utili*"[Title/Abstract] OR hospitaliz*[Title/Abstract] OR hospitalis*[Title/Abstract] OR length of stay[Title/Abstract] OR cost[Title/Abstract] OR ((inpatient OR outpatient OR emergency department) AND (days OR visits)))	228

	**Limits:** English, humans, adults, 10 years, dataset, guideline, meta-analysis, multi-center study, observational study, practice guideline, research support - Non-U.S. Gov't, review, systematic reviews, validation studies	

The search was conducted on May 9, 2019
