# Supplementary material for: Systematic literature review of burden of illness in chronic inflammatory demyelinating polyneuropathy (CIDP)
Source: J Neurol. 2020 Jun 24;268(10):3706–16. doi: 10.1007/s00415-020-09998-8 (PMC8463372; doi:10.1007/s00415-020-09998-8)
Supplement: Supplementary file 2 — Supplementary material 2 (PDF 150 kb) [file 415_2020_9998_MOESM2_ESM.pdf]

Electronic Supplementary Material

Online Resource 2

Fig. 1 The incidence (A) and prevalence (B) rates reported for CIDP [1,4-6,20]

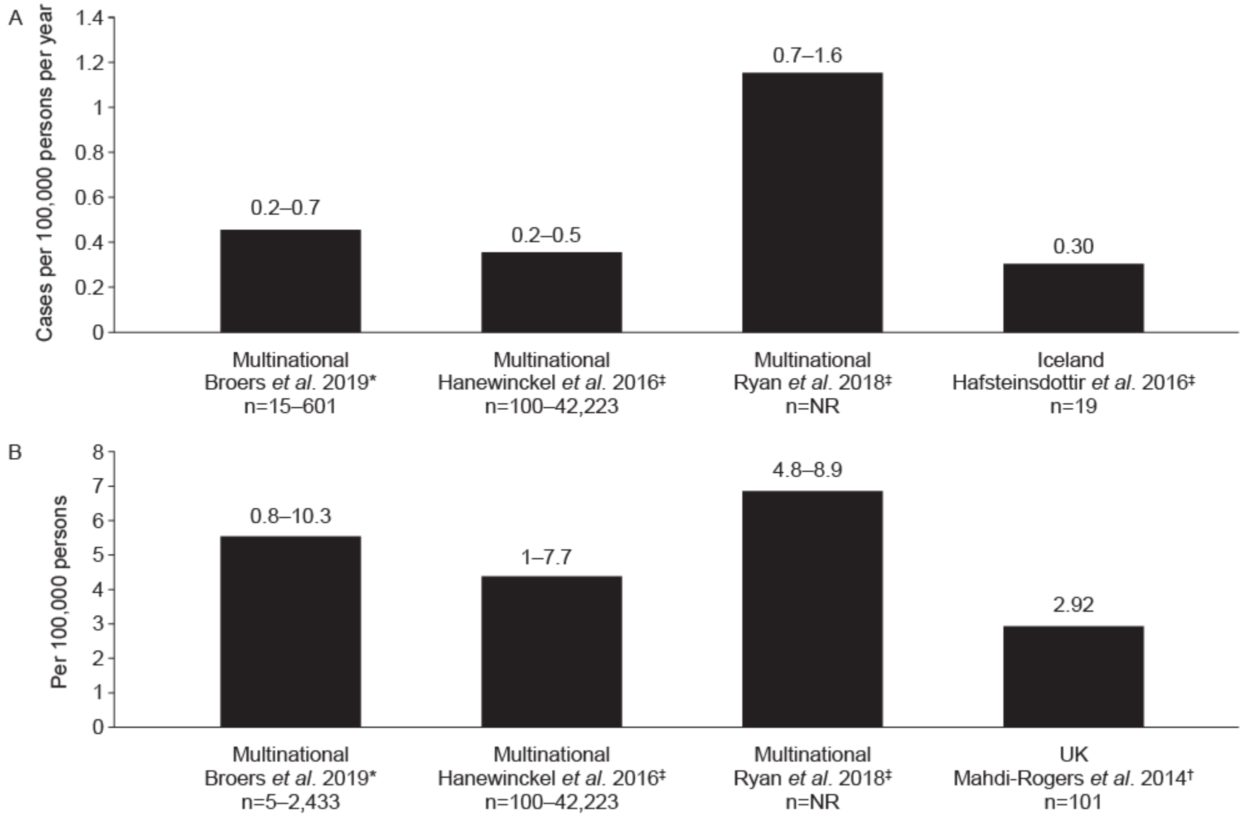

Note, ranges are included where publications spanned multiple countries and reported a range of prevalence or incidence data. *Mixed crude, standardised, and age and sex adjusted incidence/prevalence data. †Standardised incidence/prevalence data. ‡Type of incidence/prevalence data (i.e. standardised or crude) was not reported.

Multinational studies included the following countries:

- Broers *et al.* 2019: Republic of Ireland, Iceland, UK, USA, Japan, Italy, Norway, Australia and Japan
- Hanewinckel *et al.* 2016: Geographies NR
- Ryan *et al.* 2018: USA, Italy and Japan

CIDP: Chronic inflammatory demyelinating polyneuropathy; NR: Not reported.
