# Supplementary material for: Systematic literature review of burden of illness in chronic inflammatory demyelinating polyneuropathy (CIDP)
Source: J Neurol. 2020 Jun 24;268(10):3706–16. doi: 10.1007/s00415-020-09998-8 (PMC8463372; doi:10.1007/s00415-020-09998-8)
Supplement: Supplementary file 3 — Supplementary material 3 (PDF 471 kb) [file 415_2020_9998_MOESM3_ESM.pdf]

Electronic Supplementary Material

Online Resource 3

Table 2. Summary of data identified on current treatment of CIDP

Publication	Country	Patient sample	Timeframe	Treatment type(s)	Treatment response*	PROs	Tolerability
Benedetti *et al.* 2011 [51]	Italy	n=13<br>• CIDP patients who experienced partial or complete lack of efficacy of conventional therapies Patients diagnosed as per EFNS/PNS criteria	**Duration of study:** 1-5 years (range)	**Immunosuppressant -Rituximab**	NR	**Reduction of disability in QoL-related daily activities: 6 patients**<br><br>(e.g. deambulation, handling knife and fork, washing hair and doing/undoing zips)	**Overall tolerability (no. patients):**<br>• **No major AEs recorded**<br>• **Flu-like symptoms:** 1<br>• **Mild skin allergy:** 1 (responded to CS treatment)
Bril *et al.* 2018 [69]	NR	n=172<br>• Patients with assumed axonal damage (amplitude <=1) versus patients with assumed non-axonal damage (amplitude >1)	**Duration of study**: 25 weeks	**SCIG or placebo**	**Non-axonal damage relapse rates:**<br>• Placebo:73%<br>• Low-dose IVIG:39%<br>• High-dose IVIG:19%<br>**Axonal damage relapse rates:**<br>• Placebo IVIG: 25%<br>• Low-dose IVIG: 30%<br>• High-dose IVIG: 19%	NR	NR

Publication	Country	Patient sample	Timeframe	Treatment type(s)	Treatment response*	PROs	Tolerability
Cocito *et al.* 2010 [31]	Italy	n=267<br><br>• CIDP diagnosis according to the EFNS/PNS criteria<br>• CIDP types included chronic-progressive course, relapsing-remitting course and monophasic course	**Mean:** 28 days	**IVIG, corticosteroids or plasma exchange**	**Overall responders:**<br>• Corticosteroids - 61%<br>• IVIG - 73%<br>• Plasma exchange - 48%<br>**First-line responders:**<br>• Corticosteroids- 64%<br>• IVIG - 43%<br>• Plasma exchange - 56%<br>• Total - 69%	NR	**Overall tolerability (% of patients who has side effects):**<br>• **Corticosteroids:** 12.5% (Diabetes; high BP; duodenal ulcer; osteoporosis; psychosis; obesity; MI)<br>• **IVIG:** 4% - Headache, deep vein thrombosis, MI)<br>• **Plasma exchange:** 19% - Difficult access to veins, a deficit of coagulation factors<br><br>**AEs in first-line responders:**<br>• **Corticosteroids:** 13%<br>• **IVIG:** 4%<br>• **Plasma exchange:** 25 %<br>• **Total:** 31%<br><br>**AEs in patients switching treatments:**<br>• **Corticosteroids →** IVIG: 7% (i.e. 1 patient)<br>• **Corticosteroids →** Plasma exchange: 0%<br>• **IVIG →** Corticosteroids: 7%<br>• **Plasma exchange →** Corticosteroids: 0%

Publication	Country	Patient sample	Timeframe	Treatment type(s)	Treatment response*	PROs	Tolerability
Cocito *et al.* 2018 [44]	NR	NR	NR	**SCIG or placebo after IVIG induction**	NR	**Ease of use:** 88% felt SCIG was easier to use than IVIG<br>• Significantly more subjects improved/maintained QoL health status with SCIG vs. placebo. (P-values <0.005)	NR
Doneddu *et al.* 2018 [70]	Italy	n=432<br>**Typical CIDP**: n=355 (82%)<br>• 167 (39%) patients had an initial diagnosis of atypical CIDP that in 90 (54%) patients evolved to typical CIDP<br>**Atypical CIDP**: n=77 (18%)<br>• **DADS**: n=31 (7%),<br>• **Purely motor CIDP**: n=17 (4%)<br>• **LSS or focal CIDP**: n=15 (3·5%)<br>• **Purely sensory CIDP**: n=14 (3%; including two with	NR	**IVIG and potentially additional therapies (not specified)**	Patients with DADS and LSS had a less frequent response to therapy than those with typical CIDP, mainly reflecting a less frequent response to immunoglobulins while patients with purely motor and sensory CIDP had a similarly frequent response. DADS and LSS have a less frequent response to IVIG compared to typical CIDP, possibly reflecting the presence of some differences in their underlying pathogenesis	NR	NR

Publication	Country	Patient sample	Timeframe	Treatment type(s)	Treatment response*	PROs	Tolerability
		chronic immune sensory polyradiculopathy)					
Gorson *et al.* 2013 [12]	USA	NR	NR	**Prednisone, Pulse oral dexamethasone, plasma exchange, IVIG, SCIG Pulse intravenous methylprednisolone, azathioprine, IFNß -1a, methotrexate**	• **Overall responders (based on multiple studies) to IVIG**: 45% to 70% patients<br>• **Plasma exchange (based on Cochrane review):** Improvements were observed in the mean neurologic disability scale, grip strength, clinical disability grade, and summated mean motor potential amplitudes and conduction velocities.<br>• **Comparative double-blind trial:** 48% of patients improved with intravenous methylprednisolone	NR	NR
Hartung *et al.* 2018 [49]	NR	NR	**Duration of study:** 24 weeks	**SCIG (IGPro20) or placebo (stabilisation on IVIG)**	NR	**EQ-5D score maintenance** - A higher proportion of patients treated with SC IGPro20 vs. placebo	NR

Publication	Country	Patient sample	Timeframe	Treatment type(s)	Treatment response*	PROs	Tolerability
						**Median VAS scores change in points (0.4g/kg vs. 0.2g/kg vs. placebo):** 0.0 (Q1, Q3: −7.5, 5.5) vs. −5.0 (Q1, Q3: −15.0, 6.0 points) vs. −10.0 (Q1, Q3: −25.0, 0.0 points); p<0.005, across treatment groups	
Hartung *et al.* 2018 [71]	NR	n=172	NR	**SCIG or placebo**	NR	**TSQM:** Overall satisfaction in domains of effectiveness, side effects, convenience, and overall satisfaction (ranging from 0, poorest; to 100, perfect satisfaction):<br>• **Placebo:** -11.1 points<br>• **0.2 g/kg IGPro20:** -8.3 points<br>• **0.4 g/kg IGPro20**: -5.6 points<br><br>Effectiveness domains	NR

Publication	Country	Patient sample	Timeframe	Treatment type(s)	Treatment response*	PROs	Tolerability
						• **Placebo**: -13.9 points<br>• **0.2 g/kg IGPro20:** -5.6 points<br>• **0.4 g/kg IGPro20**: -11.1 points<br><br>**WPAI: Comparison (placebo vs. 0.2 g/kg vs. 0.4g/kg IGPro20):**<br>• **Median AI loss score:** 10% vs. 0% vs. 0%<br>• **Median WI loss score:** 30% vs. 0% vs. 0%<br>• **Median WP loss score:** 22.2% vs. 2.5% vs. 0%<br>• WPAI AI, WI and WP loss scores were relatively stable in the IGPro20 groups compared with worsening on placebo at the LPDO. There was no relevant difference across groups in change score on the	

Publication	Country	Patient sample	Timeframe	Treatment type(s)	Treatment response*	PROs	Tolerability
						absenteeism domain<br>**Conclusions:** None of the treatment differences reached statistical significance	
Hughes *et al.* 2010 [52]	USA	n=67<br><br>• (IVIG)-dependent CIDP	Duration of study: 16–32 week (range)	**Intramuscular IFNß -1a or placebo**	**Intramuscular IFNß -1a** - no effect on muscle strength, ODSS, Rotterdam Handicap Scale score<br>This trial **<u>did not</u> provide evidence to support the benefit of intramuscular IFNß -1a** reported in some patients with CIDP	NR	**Overall tolerability (% patients):**<br>• **AEs:** 94% (including placebo group)<br>• **Withdrew due to AEs:** 7%<br>• **Discontinued due to drug:** 6%<br><br>**AEs (Combined IFNß -1a group vs. placebo):**<br>• **Overall AEs:** 97% vs. 86%<br>• **Flu-like symptoms:** 56% vs. 32%<br>• **Headache:** 27% vs. 27%<br>• **Fatigue:** 18% vs. 27 %<br>• **Depression:** 11% vs. 5%<br>• **Interferon beta neutralising antibodies:** 4% vs. 0%<br>• **SAEs** (Combined IFNß -1a group, none in placebo):<br>• **CIDP:** 4%<br>• **Leukopenia:** 2%<br>• **Urticaria:** 2%

Publication	Country	Patient sample	Timeframe	Treatment type(s)	Treatment response*	PROs	Tolerability
							• **Severely elevated liver enzymes (aspartate transaminase and alanine transaminase):** 2% (1 patient receiving 60 g IFN, who withdrew from the study)
Hughes *et al.* 2017 [54]	NR	n=106<br>• CIDP participants receiving IVIG or corticosteroids	Duration of study: 6 months	**IVIG or corticosteroids switching to fingolimod or placebo**	**Confirmed worsening in fingolimod vs. placebo-treated participants (hazard ratio)**<br>**Baseline INCAT score:**<br>• **INCAT<3:** 7/23 vs.7/23, 1.19 [0.42, 3.39]<br>• **INCAT=3:** 9/18 vs. 12/17, 0.62 [0.26, 1.46]<br>• **INCAT>3:** 9/13 vs. 7/12, 1.24 [0.46, 3.34])<br><br>**Confirmed worsening in fingolimod vs. placebo-treated participants (hazard ratio)**<br>**By previous treatment:**<br>• **IVIG:** 23/41 vs. 20/41,1.28 [0.70, 2.34]	NR	NR

Publication	Country	Patient sample	Timeframe	Treatment type(s)	Treatment response*	PROs	Tolerability
					• **Corticosteroids:** 2/13 vs. 6/11, 0.26 [0.05, 1.29]<br>**Duration of CIDP:**<br>• **<2 years:** 6/15 vs. 6/8, 0.52 [0.17, 1.62]<br>• **2-5 years**: 8/16 vs. 4/18, 3.07 [0.92, 10.22]<br>• **>5 years:** 11/23 vs. 16/26, 0.67 [0.31, 1.45**]**<br>**Number of worsening events in the previous 2 years:**<br>• **1 worsening event:** 9/22 vs. 12/23, 0.88 [0.37, 2.09]<br>• **2 worsening events:** 3/9 vs. 7/14, 0.68 [0.18, 2.64]<br>• **Worsening events>2:** 13/23 vs. 7/15, 1.14 [0.46, 2.87]		
Hughes *et al.* 2018 [53]	Australia, Belgium, Canada, France, Germany, Greece, Israel, Italy, Japan, Netherlands, Poland,	n=106 participants<br>• Patients with CIDP who were treated with IVIG, corticosteroids, or both before study entry	**Intervention group:** 9 months (mean)<br>**Placebo group:** 9.7 months (mean)<br>Follow-up: 12 weeks	**Oral fingolimod or placebo**	**Primary outcome:**<br>• First confirmed worsening (adjusted INCAT disability scale, treatment vs. placebo): 43% vs. 42% at the end of the study<br>**Overall outcomes (% patients, treatment vs. placebo):**	NR	**Overall tolerability (treatment vs. placebo, % patients**<br>• **Overall AEs:** 76% vs. 85%<br>• **SAEs:** 17% vs. 8%<br><br>**AEs:**<br>• **Headache:** 22% vs. 15%

Publication	Country	Patient sample	Timeframe	Treatment type(s)	Treatment response*	PROs	Tolerability
	Spain, UK, USA				• Free from worsening at discontinuation: 60% vs. 60%<br><br>**The trial ended due to futility when 44 confirmed worsening events had occurred**		• **Hypertension:** 19% vs. 2%<br>• **Pain in extremity:** 13% vs. 6%<br>• **Nasopharyngitis:** 11% vs. 13%<br>• **Paraesthesia:** 9% vs. 0%<br>• **Back pain:** 7% vs. 6%<br>• **Fall:** 7% vs. 2%<br>• **Fatigue:** 7% vs. 12%<br>• **Bronchitis:** 6% vs. 2%<br>• **Diarrhoea:** 6% vs. 4%<br>• **Dizziness:** 6% vs. 4%<br>• **γ-glutamyl transferase increase:** 6% vs. 0<br>• **Urinary tract infection:** 6% vs. 2%<br>• **Vertigo:** 6% vs. 6%<br><br>**SAEs:**<br>• **CIDP (polyradiculoneuropathy):** 4% vs. 2%<br>• **Breast cancer:** 2% vs. 0<br>• **Retroperitoneal cancer:** 2% vs. 0<br>• **GBS:** 2% vs. 0<br>• **Peripheral oedema:** 2% vs. 0<br>• **Vasculitis:** 2% vs. 0<br>• **Abdominal sepsis:** 2% vs. 0<br>• **Cellulitis:** 2% vs. 0<br>• **Bursitis:** 2% vs. 0

Publication	Country	Patient sample	Timeframe	Treatment type(s)	Treatment response*	PROs	Tolerability
							• **Gastric cancer:** 0 vs. 2%<br>• **Ankle fracture:** 0 vs. 2%<br>• **Nephrolithiasis:** 0 vs. 2%
Kaplan *et al.* 2017 [37]	USA	n= 37<br><br>• Patients that had been referred for "refractory CIDP" but had no objective response to IVIG, plasma exchange, and/or corticosteroids	**Duration of study:** 4 - 6 weeks	**IVIG, plasmapheresis, corticosteroids, cyclophosphamide, fludarabine, mycophenolate mofetil, azathioprine**	**Overall responders (measured by improvement in strength):**<br>• 13 (87%) with confirmed CIDP achieved consistent response to therapy<br><br>**Outcomes:**<br>• Improvement after increasing frequency of maintenance IVIG to bi-weekly dosing: 54% (n=13)<br>• Improvement with maintenance IVIG supplemented with corticosteroids, plasmapheresis, or mycophenolate mofetil: 14%<br>• Improvement after addition of monthly pulse cyclophosphamide infusions: 5%<br>• Symptoms relapsed despite cyclophosphamide	NR	NR

Publication	Country	Patient sample	Timeframe	Treatment type(s)	Treatment response*	PROs	Tolerability
					but stabilized after addition of fludarabine: 2.5%<br><br>**No improvement: 5%**<br>• 2 patients had a response to IVIG but relapsed before the next dose and were - treatment failures. These patients improved when begun on bi-weekly maintenance therapy alone		
Kuitwaard *et al.* 2010 [29]	Netherlands	n=27 (active but stable CIDP)<br>• Initial chronically progressive, stepwise progressive or recurrent weakness of all extremities, developing over at least 2 months, with reduced or absent tendon reflexes	**Duration of study:** 10 weeks (mean)	**IVIG - Gammagard® (freeze-dried) vs. Kiovig® (liquid)**	**Primary outcome: ODSS**<br>• Clinically insignificant treatment difference of 0.004	**SF-36 scale (Gammagard® minus Kiovig®):**<br>• **Physical functioning:** -2.1<br>• **Role-physical**: 1.8<br>• **Bodily pain:** -2.8<br>• **General health:** -1.9<br>• **Mental component summary:** 1.5	**AEs (Gammagard® vs. Kiovig®):**<br>• **Fatigue:** 10 (77%) vs. 10 (71%)<br>• **Muscle and joint ache:** 8 (62%) vs. 9 (64%)<br>• **Headache:** 8 (62%) vs. 6 (43%)<br>• **Itching:** 5 (38%) vs. 6 (43%)<br>• **Backache:** 3 (23%) vs. 6 (43%)<br>• **Dizziness:** 5 (38%) vs. 4 (29%)<br>• **Warm feeling:** 3 (23%) vs. 5 (36%)<br>• **Skin rash:** 3 (23%) vs. 5 (36%)

Publication	Country	Patient sample	Timeframe	Treatment type(s)	Treatment response*	PROs	Tolerability
							• **Pain at infusion area:** 3 (23%) vs. 4 (29%)<br>• **Cold shivers:** 6 (46%) vs. 1 (7%)<br><br>Lower occurrence of cold shivers in patients randomised to Kiovig® (p=0.03)
Kuitwaard *et al.* 2015 [32]	Netherlands, Canada	n=281<br>• Patients fulfilled the EFNS/PNS criteria for typical or atypical CIDP	**Duration of study:** 5.2 years (mean)	**IVIG, corticosteroids, plasma exchange**	• **Overall responders to IVIG:** 76%<br>• **Improved with corticosteroids:** 58%<br>• **Improved with plasma exchange:** 66% (Patients who failed to improve with IVIG)<br><br>3 patients did not respond to any of the 3 treatments	NR	NR
Kuwabara *et al.* 2015 [33]	Japan	n=100<br>• Patients fulfilling criteria for CIDP by EFNS/PNS	**Duration of study:** 76 months (median)	**Intravenous methylprednisolone pulse therapy; prednisolone; plasma exchange; azathioprine; cyclophosphamide**	**Treatment response (Typical CIDP subtype vs. MADSAM subtype; p value):**<br>• **Corticosteroids:** 83% (38/46) 72% (21/29) NS<br>• **IVIG:** 87% (26/30) 38% (6/16) <0.001<br>• **Plasma exchange:** 81% (13/16) 17% (1/6) 0.0049	NR	NR

Publication	Country	Patient sample	Timeframe	Treatment type(s)	Treatment response*	PROs	Tolerability
					• **No response to any of the above:** 0% (0/51) 23% (7/30) <0.001		
Kuwabara *et al.* 2017 [30]	Japan	n=49<br>• Patients fulfilled EFNS/PNS diagnostic criteria	**Duration of study:** 52 weeks	**IVIG**	**Sustained INCAT score improvement of 1 point or more:** 77.6% of the patients (95% CI 63.4% to 88.2%) at week 28<br>**Relapses:** 10.5% of the patients (95% CI 2.9% to 24.8%) from week 29 to week 52 (INCAT score deterioration by 1 point or more compared with that at week 28)		**Overall tolerability (% patients):**<br>• **Headache:** 32.7%<br>• **Nasopharyngitis:** 28.6%<br>• **Rash:** 12.2%<br>• **Contusion:** 10.2%<br>• **Upper respiratory tract inflammation:** 8.2%<br>• **Diarrhoea:** 6.1%<br>• **Erythema:** 6.1%<br>• **Elevation of aspartate aminotransferase:** 6.1%<br>• **Sense of fatigue:** 6.1%<br>• **Pruritus:** 4.1%<br>• **Abrasion:** 4.1%<br>• **Influenza:** 4.1%<br>• **Periodontitis:** 4.1%<br>• **Pharyngitis:** 4.1%<br>• **Inguinal hernia:** 4.1%<br>• **Nausea:** 4.1%<br>• **Elevation of alanine aminotransferase:** 4.1%<br>• **Reduction of lymphocyte count:** 4.1%<br>• **Arthropod bite:** 4.1%
Latov *et al.* 2010 [38]	USA, Canada, Germany	n=117	**Duration of study:** 24 weeks	**IVIG-C (Gamunex®)**	**ICE trial - response measured as improvement of ≥1**	NR	NR

Publication	Country	Patient sample	Timeframe	Treatment type(s)	Treatment response*	PROs	Tolerability
					**point in adjusted INCAT**<br><br>**Primary outcome:**<br>• **30 responders to IVIG-C (n = 59 in IVIG-C arm):** 47% response rate<br>• **Improved at week 6 after a second infusion:** 16 (53%)		
Leger *et al.* 2013 [40]	France, Belgium, Finland, Poland, Germany, Netherland s	n=28<br><br>• Definite or probable CIDP patients, as defined by the EFNS/PNS guidelines	**Duration of study:** 1 year	**IVIG (Privigen®, stabilised with L-proline)**	**Response defined as an improvement of ≥1 point on the adjusted INCAT): Primary endpoint:** response rate measured by adjusted INCAT score at the completion<br>• **Overall response rate:** 60.7%<br>• **IVIG-pre-treated patients vs. IVIG-naive patients response rate:** 76.9% vs. 46.7%	NR	**Overall tolerability (No/% patients):**<br>• **Headache:** 9 (32.1%)<br>• **Pain in extremity:** 6 (21.4%)<br>• **Hypertension:** 4 (14.3%)<br>• **Asthenia:** 4 (14.3%)<br>• **Leukopenia:** 4 (14.3%)<br>• **Nausea:** 3 (10.7%)<br>• **Arthralgia:** 2 (7.1%)<br>• **Influenza-like illness:** 2 (7.1%)<br>• **Haemolysis:** 2 (7.1%)<br>• **Oropharyngeal pain:** 2 (7.1%)<br>• **Contusion:** 2 (7.1%)<br>• **Rash:** 2 (7.1%)
Lewis *et al.* 2018 [72]	NR	n=245<br>• Before randomisation to SCIG or placebo, subjects underwent IVIG withdrawal and,	**Duration of study:** 24 Weeks	**IVIG – withdrawal study**	IVIG withdrawal was effective in detecting subjects not requiring IVIG therapy. For IVIG-dependent subjects, restabilisation with IGPro10 was effective		

Publication	Country	Patient sample	Timeframe	Treatment type(s)	Treatment response*	PROs	Tolerability
		upon clinical deterioration, were restabilised with IVIG			in reversing observed deteriorations within 12 weeks<br><br>**Outcomes after 10–13 weeks:**<br>• **Total patients entering restabilisation period who improved in at least 1 efficacy measure:** 91%<br>• **Patients experiencing improvements in adjusted INCAT score:** 72.9%<br>• **Patients experiencing improvements beyond study entry:** ~21%		
Mahdi-Rogers *et al.* 2009 [50]	UK	n=59<br>• Patients had chronically progressive, stepwise, or recurrent weakness of all extremities, with absent or reduced tendon reflexes and with or without sensory dysfunction	**Duration of study:** 40 weeks	**Methotrexate**	**Primary outcome: No. (%) patients with > 20% reduction in mean weekly dose of corticosteroids or IVIG**<br>• **Methotrexate (n=27):** 14 (52%)<br>• **Placebo (n=32):** 14 (44%)	NR	**Overall tolerability (No/% patients):**<br>• **Cough or shortness of breath:** 7 (22%)<br>• **Infections:** 11 (34%)<br>• **Bruises and bleeding:** 2 (6%)<br>• **Mouth ulcers:** 5 (16%)<br>• **Rash:** 1 (3%)<br>• **Nausea or vomiting:** 2 (6%)

Publication	Country	Patient sample	Timeframe	Treatment type(s)	Treatment response*	PROs	Tolerability
		developing over at least 2 months and present for at least 6 months					
Markvardsen *et al.* 2013 [14]	Denmark	n=29<br>• Patients fulfilling the EFNS/PNS criteria for CIDP<br>• Patients in maintenance therapy with IVIG that were given IVIG-responders status from their treating physicians	**Duration of study:** 20 weeks	**SCIG or placebo**	**Primary outcome:** IKS delta improved by 5.5% in SCIG<br>• **Reduction in Delta of IKS:** 3 out of 14 in the SCIG group	20 out of the 29 study subjects preferred SC to IV, due to:<br>• **Increased flexibility during daily day life**: 16 patients<br>• **More stable muscle performance**: 5 patients<br>• **Milder side effects**: 3 patients<br>• **Timesaving**: 2 patients	**Overall tolerability: SCIG-treated vs. placebo patients (No of patients):**<br>• **Redness:** 6 vs. 2<br>• **Rash:** 2 vs. 0<br>• **Itching:** 1 vs. 0
Markvardsen *et al.* 2017 [45]	Denmark	n=20<br>• Patients diagnosed with definite or pure motor CIDP, naive to immune modulatory therapy and fulfilling the EFNS/PNS criteria<br>• All participants received both	**Duration of study:** 20 weeks	**SCIG and IVIG (cross-over trial)**	NR	NR	**Number of patients who experienced AEs:**<br>• **Spontaneous and remitting severe haemolytic anaemia:** 1 (decrease in Hb of 42 g/L leading to hospitalization)<br>• **Haemolytic anaemia:** 2<br>• **Fever/chill and nausea:** 2<br>• **Mild dermatological reaction:** 2

Publication	Country	Patient sample	Timeframe	Treatment type(s)	Treatment response*	PROs	Tolerability
		therapies, 14 completing the protocol					• **Headache:** 6<br>• **Local skin reactions at the infusion sites:** 3<br>• **Nausea:** 2
Mehndiratta *et al.* 2017 [73]	UK, India	n=47<br>• (Participants had to have symptoms and signs of polyradiculoneuropathy characterized by progressive or relapsing motor and sensory dysfunction of more than one limb, of more than eight weeks' duration.)<br><br>• Cross over trial: n=18 Parallel-group trial: n=29	**Duration of study:** 4 weeks	**Plasma and sham exchange**	**Plasma exchange improvement above sham improvement: Cross over trial: 4 weeks 10 exchanges; received both treatments in randomized manner**<br>• **Disability:** 2 (95% CI 0.9-3.1)<br>• **Rapid deterioration after plasma exchange:** 8 of 12 who had improved<br><br>**Parallel-group trial: Plasma exchange (n=15) and sham (n=14)**<br>• **Impairment:** 31 points (95% CI 18-45) - maximum score 280<br><br>**Combined approach:** Results of both trials, plasma exchange produced significantly more improvement in severity of disease signs measured by neurologists than	NR	**AEs, based on observational studies:**<br>• **Difficulty with venous access**<br>• **Hemodynamic changes** occur in 3-17% of procedures<br><br>**AEs:**<br>• Citrate toxicity: 3%<br>• Vasovagal reactions<br>• Vascular access complications<br>• Cardiac arrhythmia<br>• Haemolysis<br>• Hepatitis B<br>• Fresh frozen plasma reactions<br><br>**SAEs:**<br>• In one study, 1 of 29 participants had a stroke 1 day after plasma exchange<br>• In another study 1 of 30 participants had catheter-related myocarditis, not stated whether this was after plasma exchange or sham treatment

Publication	Country	Patient sample	Timeframe	Treatment type(s)	Treatment response*	PROs	Tolerability
					sham exchange but the results reported were short-term		
Merkies *et al.* 2009 [36]	The Netherlands, France, Canada, Saudi Arabia, USA, Germany, Japan, Belgium, Finland, Poland	n=117<br><br>• Participants had a diagnosis of CIDP, progressive or relapsing motor and sensory dysfunction of at least one limb resulting from neuropathy over at least 2 months before study	**Duration of study:** 24 weeks	**IVIG vs. placebo**	NR	**LSM change from baseline between IVIG-C vs. placebo (Improvement)**<br>• **Physical functioning:** 15.6 vs. 3.8<br>• **Role-physical:** 21.0 vs. 5.2<br>• **Bodily pain:** 8.7 vs. 0.6<br>• **General health:** 7.3 vs. 0.9<br>• **Vitality:** 9.1 vs. 3.6<br>• **Social functioning:** 13.2 vs. 2.4<br>• **Role-emotional:** 12.3 vs. 5.3<br>• **Mental health:** 8.2 vs. 0.4	NR
Merkies *et al.* 2019 [41]	France, Canada, Saudi Arabia, USA, Germany, Japan, Belgium,	n=28 patients (PRIMA study)<br><br>• IVIG pre-treated, n = 13; previously untreated, n = 15	**Duration of PRIMA study:** 25 weeks<br>**Duration of PATH study:** 13 weeks	**IVIG - IGPro10 (Privigen®) and SCIG (Hizentra®)**	**INCAT response rate**<br>**PRIMA IVIG (at Week 25):**<br>• **Pooled cohort (n = 235):** 71.5% (95% CI: 65.9–77.3)	NR	• **Overall tolerability (number of events):** 108 AEs occurred in 22 (78.6%) subjects (0.417/infusion)<br><br>• **IVIG pre-treated PRIMA subjects (n = 13) :**41 AEs occurred

Publication	Country	Patient sample	Timeframe	Treatment type(s)	Treatment response*	PROs	Tolerability
	Finland, Poland	n=207 (PATH study), IVIG pre-treated					in 10 (76.9%) subjects (0.366/infusion)<br><br>• **Treatment-naïve subjects:** 67 AEs reported in 12 (80.0%) subjects (0.456/infusion)<br><br>**Safety population (n = 207):**<br>• **PATH (SCIG):** 284 AEs in 100 (48.3%) subjects (0.175/infusion)<br><br>**Headache: Most frequent AE**<br>• **PRIMA (IVIG):** 9 (32.1%) PRIMA subjects (4 pre-treated, 5 treatment-naïve subjects)<br>• **PATH (SCIG):** 34 (16.4%) PATH subjects (overall 42/235 subjects [18.3%])<br><br>**Causally related serious AEs:**<br>• **PRIMA (IVIG):** 2 subjects (haemolysis)<br>• **PATH (SCIG):** 7 subjects (hypersensitivity, pulmonary embolism, increased blood pressure, exacerbation

Publication	Country	Patient sample	Timeframe	Treatment type(s)	Treatment response*	PROs	Tolerability
							of CIDP, respiratory failure, rash, migraine)<br><br>**ADRs in pooled population:** 0.144 ADRs per infusion, frequent ADRs were headache, nausea, hypertension, and haemolysis<br>• **PRIMA (IVIG):** 20 subjects (71.4%) had 71 ADRs<br>• **PATH (SCIG):** 85 subjects (41.1%) had 200 ADRs
Mielke *et al.* 2019 [39]	Germany, Canada, Saudi Arabia, The Netherlands, US, Japan	n=245<br>• Adult subjects with definite or probable CIDP, all being treated with IVIG before enrolment were eligible for this study	**Duration of study:** 17 weeks	**IVIG - IGPro10 (Privigen®) – withdrawal study**	**Improvements after three doses of IGPro10:** 99%<br><br>**IVIG withdrawal:** Effective in detecting ongoing IG G dependency with a small risk for subjects not returning to their baseline 17 weeks after withdrawal<br><br>**Of patients treated with IVIG in the restabilisation period:**<br>• **Did not improve:** 17%<br>• **Stable:** 83%	NR	• **IVIG restabilisation period (n=100/207):** 48.3% experienced 284 AEs<br>– **Causally related AEs:** 28% of the subjects<br>– **Common AEs: >5 % subjects**<br>▪ Headache<br>▪ Nasopharyngitis<br>▪ Nausea<br>– **Serious AEs:** 7 serious AEs experienced by subjects<br>▪ Allergic reaction<br>▪ Pulmonary embolism<br>▪ Increase in diastolic blood pressure<br>▪ Exacerbation of CIDP<br>▪ Worsening of respiratory failure

Publication	Country	Patient sample	Timeframe	Treatment type(s)	Treatment response*	PROs	Tolerability
							▪ Rash<br>▪ Worsening of migraine<br><br>• No unexpected AEs associated with IGPro10<br>• Headache and nasopharyngitis were the most frequently reported AEs during restabilisation<br>• AEs deemed causally related were mostly mild or moderate
Nobile-Orazio *et al.* 2012 [74]	Italy	n=45<br>**Patients**:<br>• Definite, typical CIDP according to the EFNS/PNS criteria<br>• Had some disability (scoring 2 or more on either the ONLS or the modified Rankin scale)<br>• Were in an active or stationary phase but not in remission compared with the last	**Duration of study: 6 months**<br>**Follow-up: 6 months**	**IVIG, intravenousmethyl prednisolone**	**Post-study follow-up information on subject who did not reach stability (n=16)**<br>• **Improved to baseline clinical status:** 9 (56%)<br>• **Did not improve from baseline:** 7 (44%)<br>• **During the restabilization period (n=207)**<br>• **Improved in at least one of the predefined outcome measures:** 188 (91%)<br><br>**“CIDP stability” = no relevant change in**	NR	• No unexpected AEs associated with IGPro10<br>• Headache and nasopharyngitis were the most frequently reported AEs during restabilization<br>• AEs deemed causally related were mostly mild or moderate

Publication	Country	Patient sample	Timeframe	Treatment type(s)	Treatment response*	PROs	Tolerability
		available assessment<br>• Were without improvement in the ONLS and modified Rankin scale scores between the screening and inclusion visits			**INCAT score at last two restabilization visits and at least the same total score as at screening**		
Nobile-Orazio *et al.* 2018 [34]	NR	n=368<br>(305 patients fulfilling the EFNS/PNS criteria)<br>• **Typical CIDP**: n=368 (81%)<br>• **Atypical CIDP**: n=84 (19%)	**Duration of study:** 2 years	**IVIG, corticosteroids, plasma exchange, immune suppressant**	**Improvement after therapy (combined for all therapies):** 87%<br>**Response to therapies:**<br>• **IVIG:** 74%<br>• **Corticosteroids:** 52%<br>• **Plasma exchange:** 53%<br>• **Immunosuppressants**: 37% with Rituximab being the most frequently effective (70%)	NR	NR
Pasnoor *et al.* 2017 [35]	NR	**n=38**<br>• **CIDP EFNS definite:** n=28<br>• **EFNS probable:** n=5 (13.16%)<br>• **AAN:** n=10 (26.3%)	**Not applicable:** Retrospective data analysis	**IVIG, intravenous or oral corticosteroids, mycophenolate mofetil, plasma exchange**	**Treatment responders in EFNS definite/probable group (responders/n) defined response based on treating physician's impression of change, patient–reported functional**	NR	NR

Publication	Country	Patient sample	Timeframe	Treatment type(s)	Treatment response*	PROs	Tolerability
		• **INCAT criteria:** n=20 (52.6%)<br>• **Saperstein criteria:** n=20 (52.6%)			**improvement or one-point grade change in the MRC grade)**<br>• **IVIG:** 20/22<br>• **Intravenous or oral corticosteroids:** 5/8<br>• **Mycophenolate mofetil:** 2/3<br><br>**Treatment responders in AAN group responders/n)**<br>• **IVIG:** 8/9<br>• **Intravenous or oral corticosteroids:** 2/3<br>• **Mycophenolate mofetil:** 1/1<br><br>**Treatment responders in INCAT group (responders/n)**<br>• **IVIG:** 10/15<br>• **Intravenous or oral corticosteroids:** 5/7<br>• **Mycophenolate mofetil:** 1/2<br><br>• **Half to two-thirds of patients responded to plasma exchange based on different criteria**<br>EFNS 2010 criteria are most sensitive for		

Publication	Country	Patient sample	Timeframe	Treatment type(s)	Treatment response*	PROs	Tolerability
					the clinical diagnosis of CIDP		
Querol *et al.* 2013 [42]	Spain	n=86<br>• Patients meeting the EFNS/PNS criteria	**Duration of study:** 48 weeks	**IVIG, immunosuppressant treatment, prednisone**	The percentage of responders was higher in patients who received concomitant immunosuppressant treatment than in patients who received only IVIG (71.4% vs. 44.0%; OR 3.18; P50.01) at the mid-term visit<br><br>In the long-term analysis, 22 (25.6%) patients were in remission, 56 (65.1%) were stable, and 8 (9.3%) did not respond to treatment.	NR	NR
Querol *et al.* 2014 [75]	US, Spain	**CIDP patients**: n=61<br>• **Patients meeting the EFNS/PNS criteria**: n=53<br>• **IVIG-resistant patients from a Spanish National Registry (CIBERNEDCIDP)**: n=8	**Not applicable:** only in vitro samples taken, and MRI performed	**IVIG, control**	**IGG4 antibodies:** 4 patients with NF155 antibodies were non-responders<br>**Refractory to IVIG**<br>• 2 patients with NF155 antibodies developed severe polyradiculoneuropathy with a predominant distal weakness that was refractory to IVIG<br>• 8 additional patients with IVIG-refractory CIDP	NR	NR

Publication	Country	Patient sample	Timeframe	Treatment type(s)	Treatment response*	PROs	Tolerability
					were then identified from a national database, 2 of them with the same clinical features also had NF155 antibodies<br><br>**Patients with CIDP positive for IGG4 NF155 antibodies:**<br>• Constitute a specific subgroup with a severe phenotype<br>• Poor response to IVIG<br>• Disabling tremor		
Shebl *et al.* 2018 [47]	NR	n=207<br>• IVIG re-stabilisation comprised an initial dose of 2g/kg followed by 3–4 doses of 1g/kg at 3-week intervals. Subjects were then randomised to weekly SCIG maintenance therapy or placebo	**Duration of study:** 24 weeks	• **IVIG: an initial dose**<br>• **SCIG maintenance therapy or placebo**	• **Subjects, re-stabilised on IVIG:** 83%<br>• **Did not relapse SCIG 0.4g/kg vs. 0.2g/kg vs. placebo (% subjects):** 81% vs. 67% vs. 44%<br>• **Did not relapse SCIG 0.4g/kg vs. 0.2g/kg vs. placebo (% subjects):** 81% vs. 67% vs. 44%	NR	**Overall tolerability**<br>• **Headaches:** 65%<br>• **Local reactions:** 95%<br><br>**IVIG vs. SCIG AEs (number of events)**<br>• **Haemolysis:** 9 (all non-serious and resolved without transfusion) vs. 0<br>• **Thromboembolic events, renal failures or deaths:** 0
Spillane *et al.* 2017 [76]	UK	n=67	**Duration of study:** 30 months	**IVIG**	NR	NR	**Thromboembolic events (number of events):**<br>• **MIs:** 6

Publication	Country	Patient sample	Timeframe	Treatment type(s)	Treatment response*	PROs	Tolerability
							• **CVAs:** 2<br>• **TIA:** 1<br>• **DVT:** 1<br>• **PuE:** 1<br>• **SVC:** 1; obstruction due to central line thrombosis<br>IVIG cohort vs. population-based estimates from UK hospital coding records (per 1000 patient-years)<br>• **TEE incidence (95% CI):** 42.1 (18.6-67.1) vs. 15.29 (15.25-15.33)<br>• **IVIG-ATE incidence:** 32.1(11.1-53.1) vs. 12.9 (12.8-13.0)<br>• **IVIG-VTE:** 10 (1.4-22.8) vs. 2.37 (2.36-2.39)<br><br>**Correlations with events:**<br>• **Age (p=0005):** was higher than those who did not have an event<br>• **QRISK2 score:** p=0.01 - was higher<br>• **Dose/ day:** p=0.008) was lower
Stangel *et al.* 2013 [77]	Germany	n=21	**Duration of study:** 2 years	**IVIG, SCIG**	NR	**Patients treated with IG response to how you would describe your health (SF-36)**<br>• **Good:** 17%	NR

Publication	Country	Patient sample	Timeframe	Treatment type(s)	Treatment response*	PROs	Tolerability
						• **Moderate:** 50%<br>• **Poor:** 33%<br>• **QoL reduced in all domains**: social functional capability, physical functional capability and physical role function	
Topa *et al.* 2017 [48]	NR	n=13<br>• **Sex:** 8 males and 5 females mean age: 58 ± 11.4 years mean age at onset: 45.9 ± 12.4 years.<br>• **Disease duration:** 11.9 ± 8.3 years.<br>• Patients were previously responders to IVIG	**Duration of study:** 2 years	**Initial dose of IVIG. then SCIG (continuous regimen and pulsed regimen**)	**Pulsed SCIG treatment: Responded to SCIG similarly to IVIG:** 4/8 (50%)<br>**Worsened:** 3 (37.5%) - needed to be treated again with IVIG<br>**Stopped any therapy:** 1 (12.5%)<br><br>**A continuous regimen of SCIG: Clinically stable throughout the follow-up period:** 5/5 (100%)	NR	SCIG was well tolerated and no patients reported AEs
Van Lieverloo *et al.* 2018 [15]	Italy, The Netherlands, Serbia	n=125<br>• Treatment naïve CIDP patients	**Duration of study:** 4.5 years (mean)<br><br>**Prednisolone:** 15 months (median)<br>**Dexamethasone:** 5 months	**Prednisolone, pulse dexamethasone, pulse intravenous methylprednisolone**	**Improved after corticosteroid treatment:** 60% (95% CI 51–69%)<br>**Responders to corticosteroid treatment:** 60% (95% CI 51–69%)	NR	• **AEs were reported in 10 (8%) patients:** Prednisolone group: 9; Dexamethasone group: 1<br>• **AEs included hypertension, diabetes mellitus de novo, glaucoma, depression,**

Publication	Country	Patient sample	Timeframe	Treatment type(s)	Treatment response*	PROs	Tolerability
			**Intravenous methylprednisolone:** 42 months (median)		• **Prednisolone:** 57%,<br>• **Dexamethasone:** 68%,<br>• **Methylprednisolone treatment:** 57%<br>Response to steroids was seen in 3 of 12 (25%) patients with multifocal CIDP<br>**Remission after corticosteroid treatment:** 61%<br>• 20/29 (69%) who experienced a relapse, did so in the first 6 months after treatment withdrawal<br><br>The probability of responders reaching 5-year remission was 55% (95% Cl 44–70%), with no difference between the three groups		**cushingoid appearance, and gastrointestinal complaints**<br>• **SAEs occurred in 2 patients in the prednisolone group**
Van Schaik *et al.* 2010[16]	The Netherlands, UK	n=40<br>• Patients had been newly diagnosed as having definite or probable CIDP according to the European neuromuscular centre	**Duration of study:** 1 to 32 weeks<br>**Group 1: Dexamethasone:** 4 days<br>**placebo:** 24 Days<br>**repeated for** 6 cycle	**Dexamethasone, prednisolone, placebo**	**After 12 months, 16 patients were in remission:**<br>• **High-dose dexamethasone group:** 10<br>• **Prednisolone group:** 6<br>**(OR 1.2, 95% CI 0.3–4.4)**	NR	• **Most AEs were minor** and did not differ substantially between treatment groups<br>• **Sleeplessness and Cushing's face** occurred more often in the prednisolone group

Publication	Country	Patient sample	Timeframe	Treatment type(s)	Treatment response*	PROs	Tolerability
		diagnostic criteria.	**Group 2: Prednisolone:** 32 weeks				
Van Schaik *et al.* 2018[78]	NR	n=82	**Duration of study:** 48 weeks	**IVIG**	**The relapse rates depending on previous treatment in PATH:**<br>• **0.4g/kg group:** 4.8–13.6%<br>• **0.2g/kg group:** 40.0–50.0%<br>After dose reduction from 0.4g/kg to 0.2g/kg, 52% of patients worsened (32% who completed PATH without relapse on either dose) of which 89% improved after re-initiation of the 0.4g/kg dose.	NR	• **Patients with AEs:** 76% (62/82) had 180 AEs (AEs)<br>• **Percentage of AEs that were mild or moderate:** 93% of AEs<br>• **Percentage of AEs that were serious:** 0%
Van Schaik *et al.* 2018 [46]	The Netherlands, Canada, Germany, USA, Japan	n=172<br>• Patients had been diagnosed with definite or probable CIDP according to the EFNS/PNS criteria and received their last IVIG treatment at least within 8 weeks before enrolment	**Duration of study**: 24 weeks	**SCIG low-dose group; SCIG high-dose group, placebo**	**Relapse or withdrawal in the intention-to-treat set:**<br>• **Placebo:** 36 (63% [95% CI 50–74])<br>• **Low-dose:** 22 (39% [27–52])<br>• **High-dose:** 19 (33% [22–46]) (p=0.0007)	NR	NR

Publication	Country	Patient sample	Timeframe	Treatment type(s)	Treatment response*	PROs	Tolerability
Wietek *et al.* 2018 [43]	Austria	n=58	**Duration of study:** 9.7 months (mean)	**IVIG (Octagam®)**	**Clinical appearance since last observation (mean: every 9.7 months):**<br>• **81.1% (142/175)** assessed the patients as stable<br>• **16.6% (29/175)** of observations showed an improved clinical appearance<br>• **2.3% (4/175)** of the observation periods resulted in deteriorations<br>In two studies that included 28 CIDP patients, physicians rated the influence as beneficial and as unchanged in 14 (50%) patients each.	NR	• **Overall AEs:** 5 (0.61% of infusions in this cohort)<br>• **SAEs:** 1

Note, where publications provided multiple analyses of the same study, duplicate data were only reported in the first instance. *Including rates of response, improvement, remission, relapse, and worsening.

AAN: American Academy of Neurology; ADR: Adverse drug reactions; AE: Adverse event; AI: Activity impairment; ATE: Arterial thromboembolic events; BP: Blood pressure; CIDP: Chronic inflammatory demyelinating polyneuropathy; cIKS: Combined isokinetic muscle strength; CI: Confidence interval; CVA: Cerebrovascular accident; DADS: Distal acquired demyelinating symmetric neuropathy; DVT: Deep vein thrombosis; EFNS/PNS: European Federation of Neurological Societies/Peripheral Nerve Society; GBS: Guillain–Barré syndrome; ICE: Immune Globulin Intravenous CIDP Efficacy; IFNß: Interferon beta; IGG4: Immunoglobulin G4; INCAT: Inflammatory Neuropathy Cause and Treatment; IVIG: Intravenous immunoglobulin; LDPO: Last dose post observation; LSS: Lewis-Sumner syndrome; LSM: Least square mean; MADSAM: Multifocal acquired demyelinating sensory and motor neuropathy; MI: Myocardial infarction; NF155: Neurofascin 155; NR: Not reported; ODSS: Overall Disability Sum Score; ONLS: Overall Neuropathy Limitations Scale; OR: Odds ratio; PATH: Polyneuropathy and Treatment with Hizentra; PRIMA: Privigen Impact on Mobility and Autonomy; PuE: Pulmonary embolism; QoL: Quality of life; SAE: Serious adverse event; SCIG: Subcutaneous immunoglobulin; SF-36: Short Form 36; SVC: Superior vena cava; TEE: Thromboembolic events; TIA: Transient ischaemic attack; TSQM: Treatment Satisfaction Questionnaire for Medication; VAS: Visual analogue scale; VTE: Venous thromboembolic events; WI: Work impairment; WP: Work productivity; WPAI: Work Productivity and Activity Impairment
