# Supplementary material for: Systematic literature review of burden of illness in chronic inflammatory demyelinating polyneuropathy (CIDP)
Source: J Neurol. 2020 Jun 24;268(10):3706–16. doi: 10.1007/s00415-020-09998-8 (PMC8463372; doi:10.1007/s00415-020-09998-8)
Supplement: Supplementary file 4 — Supplementary material 4 (PDF 226 kb) [file 415_2020_9998_MOESM4_ESM.pdf]

Electronic Supplementary Material

Online Resource 4

Table 3. Summary of data identified related to economic burden of CIDP

Publication	Country	Patient sample	Total cost of illness	Direct costs	Indirect costs	Health resource utilisation
Alexandrescu *et al.* 2014 [24]	UK	n=15<br>• Sample derived from the UKROC database, which collates the national clinical dataset for in-patient specialist rehabilitation in England	NR	NR	NR	**Estimated care requirements**<br>**Upon patient admission to neurorehabilitation centres (hours/week, mean):** 35.0<br>**Upon patient discharge from neurorehabilitation centres (hours/week, mean):** 22.5

Publication	Country	Patient sample	Total cost of illness	Direct costs	Indirect costs	Health resource utilisation
Krishnarajah *et al.* 2017 [59]	US	Cases: n=790<br><br>Controls: n=790<br><br>• Sample derived from QuintilesIMS PharMetrics Plus Health Plan Claims Database	**Median, CIDP vs. controls without CIDP**<br><br>**Total healthcare costs six-months prior to diagnosis:** $4,751 vs. $2,209 (p<0.0001)<br><br>**Total healthcare costs two years after diagnosis:** $47,827 vs. $5,823 (p<0.0001)	**CIDP-related therapy costs (% of total healthcare costs):** 51.4%	NR	NR

Publication	Country	Patient sample	Total cost of illness	Direct costs	Indirect costs	Health resource utilisation
Divino *et al.* 2018 [56]	US	n=790<br><br>• Sample derived from US managed care enrolees identified in IQVIA RWD Adjudicated Claims database based on >1 medical claim with a diagnosis for CIDP	**Mean, CIDP vs. controls without CIDP (p<0.0001)**<br><br>**Two-year total costs:** $116,330 vs. 15,586	**Mean, CIDP vs. controls without CIDP (p<.00001)**<br><br>• **Outpatient Pharmacy 2-year costs:** $11,186 vs. $3,953<br>• **Medical 2-year costs:** $105,144 vs. $11,633<br>• **Inpatient 2-year costs:** $16,357 vs. $2,862<br>• **ER 2-year costs:** $1,188 vs. $468<br>• **Physician office 2-year costs:** $5,122 vs. $2,208<br>• **Outpatient surgery 2-year costs:** $3,204 vs. $1,157<br>• **Lab/pathology 2-year costs:** $2,907 vs. $1,903<br>• **Outpatient ancillary, radiology and HCPCS drugs 2-year costs:** $76,366 vs. $4,292<br><br>**Total CIDP therapy:** $59,619	NR	**CIDP patients vs. controls without CIDP (mean), (p<0.0001)**<br><br>• **≥ 1 Hospitalisation:** 26.2% vs. 9.0%<br>• **Outpatient prescription fills:** 62.8 vs. 32.0<br>• **Physician office visits:** 34.7 vs. 13.0<br>• **ER visit:** 42.2% vs. 21.9%<br>• **Outpatient surgical visit:** 77.1% vs. 45.9%<br>• **≥1 neurologist office visit:** 67.0% vs.3.9%<br>• **Physical therapy visits:** 14.7% vs. 2.8%

Publication	Country	Patient sample	Total cost of illness	Direct costs	Indirect costs	Health resource utilisation
Guptill *et al.* 2014 [57]	US	n=73<br><br>• Sample included patients with a diagnosis of CIDP between January 1, 2011, and December 31, 2011 whose primary health plan was eligible for the AHS care management program<br>• Patients must have had at least 1 health insurance claim during the study period	NR	**Mean cost, per patient per year**<br><br>• **Health plan paid costs**: $56,953 (95% CI $36,457–77,450)<br>• **Medical costs (mean):** $25,546 (95% CI $11,419–$38,690)<br>• **Pharmacy claims (mean):** $31,899 (95% CI$17,197–$46,600)<br>• **Nerve conduction and EMG studies (mean):** $913<br>• **Healthcare costs women vs. men (mean):** $47,094 vs. $64,648 (P=0.38)<br>• **Health plan paid costs for patients treated with IVIG:** $108,016<br>• **Cost per IVIG infusion:** $8,598 ± $476<br><br>**Costs for IVIG administration according to setting**<br><br>• **At home:** $9,720<br>• **Outpatient:** $9,718<br>• **Clinic:** $2,610<br><br>**Distribution of health plan costs:**	NR	**IVIG infusion setting:**<br>• **Home:** 72%<br>• **Physician office:** 16%<br>• **Outpatient hospital:** 11%<br>• **Other place of service:** 1%

Publication	Country	Patient sample	Total cost of illness	Direct costs	Indirect costs	Health resource utilisation
				• **Injectable drugs billed using HCPCS codes**: 51%<br>• **Inpatient hospital:** 18%<br>• **Outpatient hospital:** 16%<br>• **Physician office:** 3%<br>• **ER:** 1%<br>• **Other:** 2%<br>• **Prescription pharmacy billed using NDC codes:** 6%<br>• **Home:** 3%		

Publication	Country	Patient sample	Total cost of illness	Direct costs	Indirect costs	Health resource utilisation
Le Masson *et al.* 2018 [55]	France	**CIDP:** n=8<br><br>**LSS:** n=7<br><br>• Patients currently treated with IVIG in a hospital outpatient setting were recruited for a before–after study from two tertiary referral care centres	**Annual costs (extrapolated)**<br><br>**Typical CIDP:**<br><br>€102,296 (Hospital IVIGs)<br><br>€47,823 (Home IVIGs)<br><br>**Atypical CIDP:**<br><br>€106,867 (Hospital IVIGs)<br><br>€62,592 (Home IVIGs)	NR	NR	NR

Publication	Country	Patient sample	Total cost of illness	Direct costs	Indirect costs	Health resource utilisation
Mahdi-Rogers *et al.* 2014 [25]	UK	n=43<br>• An epidemiological study in the administrative areas of South East London, Kent and East Sussex	**Annual costs per patient (mean):**<br>Total: £22,086	**Estimated UK annual economic costs:**<br>• **Hospital services:** £4,845,301<br>• **Primary care services:** £319,451<br>• **Social services:** £4,327,290<br>• **Treatment:** £17,489,517<br>• **Investigation:** £174,701	**Indirect cost (i.e. loss of productivity)**: £5,815<br>**Overall economic cost:** £36,0860,892<br>**Loss of productivity:** £9,704,631	**Six-month usage of healthcare and social services (% patients):**<br>• **Hospital out-patient:** 62.8%<br>• **Hospital in-patient or day case:** 39.5%<br>• **Primary care (GP):** 69.8%<br>• **Social services:** 48.9%<br>**Number of hours spent with healthcare workers (mean per month):**<br>• **Neurologist:** 0.40<br>• **General practitioner:** 0.36<br>• **Physiotherapist: 0.27**<br>• **Occupational therapist: 0.38**

Publication	Country	Patient sample	Total cost of illness	Direct costs	Indirect costs	Health resource utilisation
Mengel *et al.* 2018 [9]	Germany	n=108<br><br>• Patients who were treated in specialized neuroimmunology outpatient clinics in Marburg, Göppingen, and Düsseldorf at the time of or prior to the study	**Annual total cost (extrapolated)**: €45,000	**Direct 3-month costs (mean per patient)**<br><br>• **Total:** €9,423 (83% of all costs)<br>• **Inpatient care:** €721<br>• **Outpatient care:** €153<br>• **Stationary rehabilitation:** €35<br>• **Ancillary therapy:** €121<br>• **Special equipment:** €20<br>• **Drugs:** €7,897<br>– **IVIG:** €7,606<br>– **Cyclophosphamide:** €4<br>– **Rituximab:** €34<br>– **Azathioprine:** €4<br>– **Mycophenolate mofetil:** €30<br>– **Glucocorticoids:** €4<br>– **Other:** €115	**3-month indirect costs (mean per patient)**<br>• **Total:** €1,910 (17% of total costs)<br>• **Premature retirement:** €945<br>• **Disability:** €276<br>• **Unemployment:** €189<br>• **Sick leave:** €283<br>• **Reduction of labour time:** €217	NR

Publication	Country	Patient sample	Total cost of illness	Direct costs	Indirect costs	Health resource utilisation
Rosier *et al.* 2018 [58]	France	n=134<br>• An e-mail invitation was sent to 411 neurologists registered on a voluntary basis both from neuromuscular reference centres and outside of those centres. The neurologists provided four patient case reports (diagnosis, follow-up and treatment)	NR	NR	NR	**IVIG-associated resource use (% of cases):**<br>• **Full hospitalisation:** 43%<br>• **Day care** 49%<br>• **Home care:** 8%

AHS: Accordant Health Services; CI: Confidence interval; CIDP: Chronic inflammatory demyelinating polyneuropathy; EMG: Electromyography; ER: Emergency Room; GP: General practitioner; HCPCS: Healthcare Common Procedure Coding System; IVIG: Intravenous immunoglobulin; LSS: Lewis-Sumner syndrome; NDC: National Drug Code; NR: Not reported; RWD: Real world data; UKROC: UK Rehabilitation Outcomes Collaborative
